# Carbon substrate type shapes spatial self-organization in a multi-species biofilm community

**DOI:** 10.1093/ismejo/wrag223

**Published:** 2026-08-27

**Authors:** Deyong Zhu, Anna J Svagan, Nan Yang, Mads Frederik Hansen, Michael Kühl, Mette Burmølle

**Affiliations:** Section of Microbiology, Department of Biology, University of Copenhagen, Copenhagen 2100, Denmark; Department of Fibre and Polymer Technology, Royal Institute of Technology (KTH), SE-100 44 Stockholm, Sweden; Section of Microbiology, Department of Biology, University of Copenhagen, Copenhagen 2100, Denmark; Section of Microbiology, Department of Biology, University of Copenhagen, Copenhagen 2100, Denmark; Marine Biology Section, Department of Biology, University of Copenhagen, Helsingør 3000, Denmark; Section of Microbiology, Department of Biology, University of Copenhagen, Copenhagen 2100, Denmark

**Keywords:** multispecies biofilm, spatial self-organization, microbial community assembly, cross-feeding, 3D bioprinting, genome-scale metabolic modeling

## Abstract

Spatial organization is a defining feature of multispecies biofilms and critically influences microbial interactions and emergent community properties. However, understanding and manipulating how microbes assemble into spatially structured biofilms remains challenging because most experimental frameworks emphasize species composition and pairwise interactions, while often overlooking the spatial constraints on biofilms imposed by the environment. In this study, we focus on how carbon substrate type, distinguishing between diffusible sugars and polymeric substrates, affects biofilm self-organization in a four-member synthetic bacterial community (SynCom). Across all tested conditions, the SynCom consistently formed more biofilm biomass than any of its subsets, indicating a robust synergistic phenotype. Using chemically defined, 3D-printed hydrogel substrates with consistent physical properties, we varied carbon source composition to identify its impact on biofilm assembly. Microscopic imaging showed that carbon substrate type strongly influenced biofilm self-organization with diffusible simple carbon substrates yielding relatively intermixed communities, whereas polymer-rich carbon substrates promoted a highly structured biofilm organization characterized by the dominance and peripheral localization of polymer-degrading species. Bioinformatic analyses of carbohydrate-active enzyme repertoires and genome-scale metabolic modeling suggested bidirectional metabolite exchange among the SynCom members, which was also supported by analysis of biofilm formation in conditioned community supernatants. Together, our findings suggest carbon substrate type as an important ecological determinant of biofilm self-organization, highlighting the need to integrate environmental factors alongside species composition and metabolic potential to fully understand and manipulate natural and engineered multispecies biofilms.

## Graphical Abstract

**Graphical Abstract:**
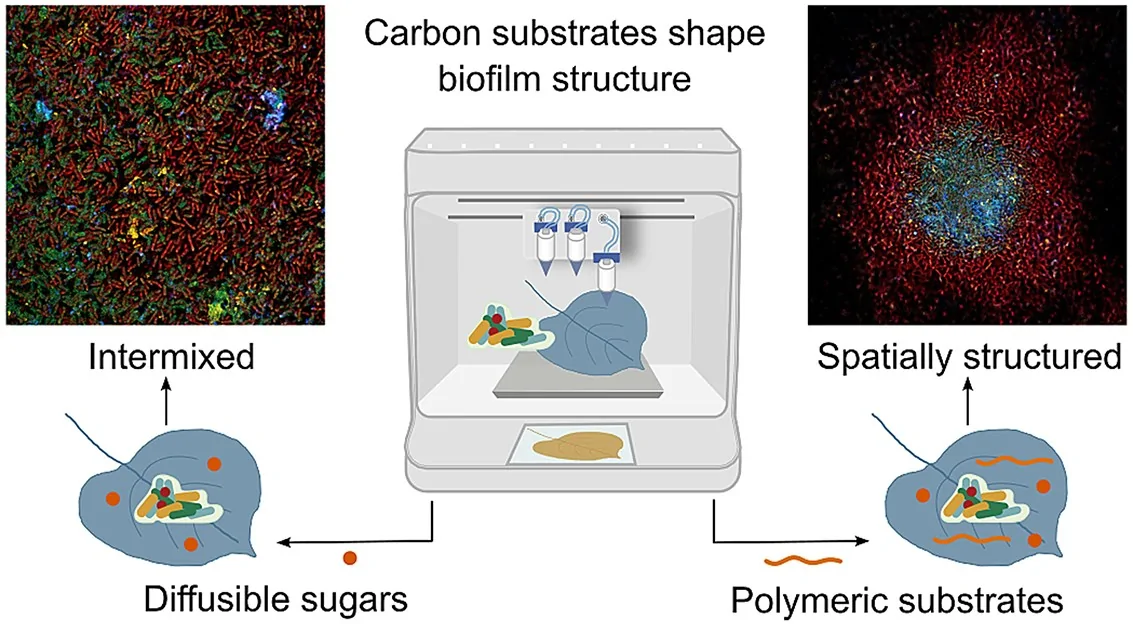

## Introduction

In natural and engineered environments, microorganisms predominantly grow as spatially structured biofilms that drive emergent ecological and functional properties collectively [1–5]. Within these assemblages, microbes exchange metabolites [6], compete for limited nutrients [7], and produce extracellular polymeric substances (EPS) [8] that confer biofilm resilience to harsh environments and stabilize the community [9–11]. Unravelling how such interactions govern biofilm architecture and function remains a central challenge in microbial ecology [12–14]. Traditionally, this challenge has been approached via studies of which species are present in the community and how they interact metabolically or chemically [15–18]. However, biofilms are fundamentally spatial systems, and recent work has highlighted that the spatial organization of species under specific conditions critically influences their interactions and expressed phenotypes [19–22].

The chemical nature of carbon substrates has long been recognized as a major determinant of microbial interactions [23–25]. For instance, carbon sources influence cross-feeding [26], niche partitioning [27], and competition-cooperation balances [28] across microbial communities. Yet, in natural habitats such as soils, plant surfaces, sediments, and decaying organic matter, microbes rarely encounter carbon substrates as homogeneous, freely diffusible solutes, which are typically employed in many biofilm model systems [29–31]. Instead, they interact with carbon resources that vary widely, with chemical and physical constraints not only in identity and concentration but also in molecular size, solubility, and accessibility [32–35]. For biofilm-associated microbes, such resource properties can generate localized gradients and impose spatial constraints that shape where cells grow, how they interact, and which metabolic strategies are favoured [9].

The spatial complexity of resource landscapes can profoundly alter microbial interactions by (i) shaping the local availability of substrates and other metabolites for cells and (ii) constraining or enhancing metabolic dependencies [36–38]. Theory and experimental observations both suggest that polymer degradation, diffusion limitation, and heterogeneous environments can reorganize species distribution and interaction networks in biofilms [39–45]. Particularly, biopolymer degradation in combination with mass-transfer limitations of metabolic substrates and products can generate microscale gradients and localized metabolic niches that select for organized communities of degraders, exploiters, and scavengers [46–49]. Despite extensive studies on polymer-degrading and cross-feeding microbial communities, we still lack a systematic understanding of how carbon substrate type and spatial distribution reconfigures the spatial organization and function of biofilms [50].

Addressing this knowledge gap requires experimental systems in which community composition is controlled, while the substrate type is systematically varied to enable quantitative investigations of how carbon substrate type alters biofilm spatial assembly. Building on methodological developments in tissue engineering, 3D printing technologies have been used to embed microbial cells within printable hydrogels, or so-called “bioinks”, to create cell-laden constructs [51–54]. These methods are now increasingly used to design and construct living materials as functional engineered systems with defined heterogeneity, e.g. controlling initial microbial placement [51–55]. However, such approaches fundamentally print the initial state, not the emergent biofilm architecture, which requires further maturation into living biofilms [56]. In these cases, the term “3D printed biofilms” is often misunderstood due to the fact that once microbes begin growing, metabolizing, and secreting EPS, these printed cell constructs quickly proliferate and remodel their own biofilm architecture, diverging from the predefined pattern far more rapidly than in 3D-printed mammalian cell constructs [57]. As a result, most prior work was not designed to assess how microbes behave when nutrients are provided in forms that mimic embedded, slowly degradable biopolymers prevalent in natural organic matter [56].

A tractable ecological test of biofilm assembly requires a platform, in which the scaffold architecture is held constant in terms of e.g. surface roughness, porosity, hydration, and diffusion constraints [58–60], while the type of carbon substrate is the variable of interest, e.g. distinguishing between freely diffusible substrates and polymeric substrates embedded in a matrix that require extracellular degradation and limit localized substrate access. Under such conditions, microbial assembly can unfold naturally through dynamic cell-to-environment and cell-to-cell interactions [61–63], enabling experimental quantification of how resource-imposed spatial constraints shape “who is where” and “how they interact”. This framework offers an opportunity to interrogate microbial spatial ecology at a level of mechanistic control not previously possible.

Based on this framework, we hypothesized that the type of carbon substrates, independent of community composition, imposes spatial constraints that drive distinct biofilm architectures and patterns of interspecies association. To test this, we utilized a four-species synthetic bacterial consortium (SynCom), originally isolated from soil and leaf litter [64], in combination with 3D-printed substrates as artificial leaves to systematically study the influence of varied carbon resources on biofilm assembly (Fig. 1A). We used extrusion-based 3D printing to formulate a reproducible hydrogel matrix, into which a carbon source can be embedded in defined formats, to let the SynCom biofilms unfold naturally. We observed synergistic biofilm formation of the SynCom across exposure to different carbon substrates, highlighting a robust phenotype not observed in monocultures or species subsets. Microscopic images demonstrated that carbon substrate type profoundly modulated the spatial biofilm organization on the printed substrates. Diffusible disaccharides yielded intermixed biofilms, whereas polymer-rich substrates induced a structured biofilm architecture centered around a dominant polymer-degrading species. Carbohydrate-Active enZymes (CAZymes) annotation and genome-scale metabolic modeling (GEMs) further predicted potential metabolic exchange networks beyond the classic degrader-exploiter-scavenger relationship in the SynCom biofilm model. Together, our findings suggest that carbon substrate type is a key determinant of emergent biofilm structure and highlight that metabolic interactions within multispecies biofilms cannot be understood independently of the spatial constraints imposed by the environment.

**Figure 1:**
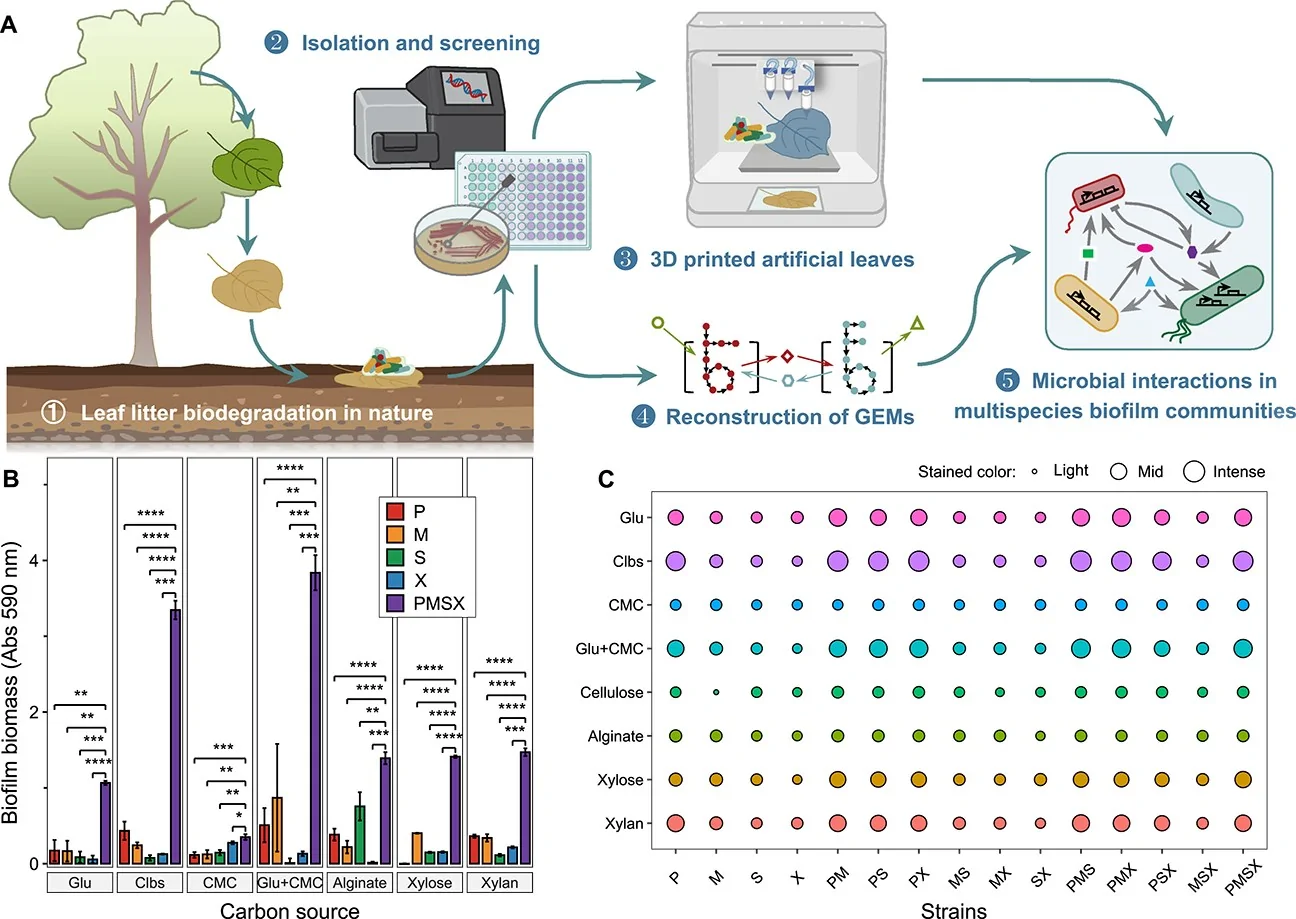
(A) A hybrid approach using 3D printed artificial leaves and genome-scale metabolic modeling (GEM) for the investigation of microbial interactions and biofilm assembly. (B) Quantification of biofilm formation of monocultures and the SynCom by crystal violet (CV) assays in media containing different carbon sources. Abbreviations for carbon sources (Glu: Glucose, Clbs: Cellobiose, CMC: Carboxymethyl cellulose) and strains (P: *P. amylolyticus*, M: *M. oxydans*, S: *S. rhizophila*, X: *S. maltophilia*, PMSX: The SynCom). Asterisks denote statistical significance of each monoculture *vs* PMSX (one-sided Welch’s *t-*test; ^*^  *P* < 0.05, ^**^  *P* < 0.01, ^***^  *P* < 0.001, ^****^  *P* < 0.0001). Error bars show standard deviation (*n* = 3). (C) Characterization of extracellular matrix production by mono-, double-, triple-, and quadruple-species biofilm colonies on agar plates using the Congo Red (CR) assay. The circles represent different carbon sources; and small circles indicate light staining, whereas large circles indicate intense staining by Congo red due to greater extracellular matrix production.

## Materials and methods

### Chemicals and bacterial species

All chemicals were purchased from Sigma-Aldrich unless otherwise noted. The *2,2,6,6*-tetramethylpiperidine-*1*-oxyl (TEMPO)-cellulose nanofiber (CNF)-based hydrogels (TEMPO-CNF, for short as CNF) were prepared as described previously in detail [65], with the exception that a M-110 EH Microfluidizer processor (Microfluidics Corporation, Westwood, MA, USA) was used. The starting pulp, a never-dried bleached sulfite pulp from spruce (<1% lignin), was a kind gift from Nordic Paper Seffle AB, Sweden.

A consortium of four bacteria, *Paenibacillus amylolyticus* (P), *Microbacterium oxydans* (M), *Stenotrophomonas rhizophila* (S), and *Stenotrophomonas maltophilia* (X), previously co-isolated from leaf litter samples in agricultural soil [8], was used as the SynCom in this study (Table S1). Bacterial isolates from −80°C preserved stock cultures were streaked on tryptic soy agar plates (containing 30 g L^−1^ tryptic soy broth (TSB) and 15 g L^−1^ agar) and incubated for 48 hours at 24°C until single colonies could be picked. Single colonies were inoculated in 5 ml TSB (30 g L^−1^) in test tubes and incubated overnight at 24°C with shaking at 250 rpm, which were referred to as overnight cultures and subsequently used in the next steps.

### Biofilm formation and growth experiments

#### Crystal violet assay

The total biomass of submerged biofilms was measured using a modified crystal violet (CV) assay [66], in which biofilms formed on the pegs of a Nunc-TSP lid system (Thermo Fisher Scientific Nunc A/S, Denmark) were quantified by CV staining. Briefly, overnight cultures of each strain were washed twice by 1× PBS and resuspended in the minimal medium M9 (prepared according to the manufacturer’s instruction and named as M9 in the following) supplemented with different carbon sources as listed in Table S2, followed by adjusting OD_600_ to 0.15. Carboxymethyl cellulose (CMC) was tested as an alternative to cellulose due to its higher water solubility. Additionally, alginate was also tested because it has been reported as a promising matrix material for 3D printing [67]. The synthetic communities were prepared by mixing the cultures at equal volumes. An aliquot of 150 μl of each culture or combination was added per well in a 96-well microtiter plate with three replicates per sample. After 24 h incubation at 24°C under static condition, the peg lid was transferred sequentially to three washing trays containing 1× phosphate-buffered saline (PBS) to wash off planktonic cells, followed by staining the biofilms formed on the pegs in a 96-well microtiter plate containing 180 μl of 1% (w/v) CV solution per well. After 20 min of staining, the peg lid was rinsed by 1× PBS five times and then placed into a new microtiter plate with 200 μl of 96% ethanol per well. After standing 25 min to allow the bounded CV molecules to dissolve into ethanol, the absorbance of CV in ethanol was measured at 590 nm by the plate reader (ELx808, BioTeck Instrument, VT, USA). Dilution of the CV-ethanol suspension was applied if the absorbance value was above 2.

#### Congo Red assay

The extracellular matrix of mono-, double-, triple-, and quadruple-species biofilm colonies was characterized by the modified Congo Red (CR) assay [68]. CR-agar plates were prepared by mixing 1.5% (w/v) Difco Agar Noble (BD, France), 40 μg ml^−1^ CR, and M9 supplemented with carbon sources as listed in Table S2. Mono-species and combinations of bacterial cultures were prepared as previously described in the CV assay, except that the OD_600_ was adjusted to 0.3. An aliquot of 3 μl from each culture was inoculated on the CR-agar plates. All plates were incubated at 24°C for 3 days for colony growth and CR binding to polysaccharides and amyloid fibers produced as part of EPS during biofilm formation [69]. Images were acquired by a Canon EOS 700D camera and digitally processed by ImageJ (https://imagej.nih.gov/ij/) to quantify the color changes of the colony zone as compared to the background.

### Rheological property measurement

The rheological property of the formulated hydrogel-based 3D printing materials was measured on a hybrid rheometer (Discovery HR-3, Thermal Analysis Instruments, DE, USA) equipped with a 40 mm parallel plate geometry and a measurement gap of 200 μm. Shear rates in the range of 0.1 to 1000 s^−1^ at a frequency of 1 Hz were used to determine the viscoelastic range of the 3D printing materials.

### 3D printing artificial leaves

The formulation and carbon source concentrations of 3D printing materials used in this study are listed in Table S3. The printing models of the testing grid and artificial leaves (original images from a lime tree leaf) were generated in CAD software (Fusion 360, Autodesk Inc., CA, USA). The printable files were produced and validated using HeartWare (Cellink Inc., Sweden). All models were printed by a commercial 3D bioprinter (BioX G2, Cellink Inc., Sweden) equipped with a 22G/0.41 mm nozzle. Printing parameters, including pressure, traveling speed, and infill rate/geometry, were optimized based on the material’s printability. Typical parameters were 15 kPa (pressure), 6 mm s^−1^ (traveling speed), and 95% infill with honeycomb-like infill geometry for the CNF-based hydrogels. The printed substrates were post-treated in a laminar flow hood for 4 h for partial dehydration prior to inoculation.

### Biofilm formation on the printed substrates containing different carbon sources

Mono-species bacterial cultures and the SynCom were prepared as described above for the CR assay. The microbial cell suspensions were evenly inoculated onto the artificial leaves containing different carbon sources, followed by incubation in petri dishes sealed with gas-permeable but water vapor-proof membranes (Breathe-Easy sealing membrane, Sigma-Aldrich Labware, Diversified Biotech, MO, USA) under controlled temperature and humidity (24°C, 60%RH).

### Biofilm characterization by CLSM

#### Live/dead staining

Biofilm samples were washed twice with 0.9% NaCl and then stained by the FilmTracer LIVE/DEAD Biofilm Viability kit (Invitrogen; Thermo Fisher Scientific, OR, USA) according to the manufacturer’s instructions. After removal of the washing buffer residues, biofilms formed on artificial leaves were *in situ* characterized by CLSM (Zeiss LSM 800 Airyscan) with an LD Plan-Neofluar 40×/0.6 objective using the Quick-LUT function to set the pixel saturation limits. The maximum excitation/emission wavelengths for SYTO 9 and propidium iodide (PI) were 482/500 and 490/635 nm, respectively. Images with Z-stacks (layer distance 1 μm) were processed by the free software Imaris Viewer (version 10.0.1, Bitplane AG, Belfast, UK) and Comstat2 (version 2.1, https://www.comstat.dk/) [70].

#### Fluorescence in situ hybridization

A protocol described previously by Liu et al. [71] was applied with some modifications. Briefly, biofilm samples on artificial leaves were fixed in 4% (w/v) paraformaldehyde in 1× PBS overnight at 4°C. After washing twice in 1× PBS (2 min each), the samples were incubated in the permeabilization solution containing 1 mg ml^−1^ lysozyme at room temperature for 10 min. After another two rounds of washing in 1× PBS (2 min each), the samples were dehydrated by three steps of washing with 50%, 75%, and 100% ethanol, respectively, for 3 min each at room temperature. The hybridization procedure with the target fluorochrome-labeled oligonucleotides probes (Table S4) was carried out in a freshly prepared stringency hybridization buffer (0.9 M NaCl, 20 mM Tris/HCl (pH 7.2), 9% formamide, 0.05% (w/v) sodium dodecyl sulfate (SDS), and probes at a final concentration of 5 ng each per μl) at 46°C for 2.5 h. Finally, the samples were washed twice with a prewarmed washing buffer (20 mM Tris/HCl (pH 7.5), 102 mM NaCl, and 0.01% (w/v) SDS) and incubated at 48°C for 15 min. The samples were ready for imaging by the Zeiss LSM 800 CLSM with a Plan-Apochromat 63×/1.4 oil DIC M27 objective (Excitation/Emission: 402/455 nm (X), 492/518 nm (S), 548/561 nm (M), and 650/673 nm (P)) after rinsing with ddH_2_O. Single-channel fluorescence *in situ* hybridization (FISH) images were converted to per-pixel maximum intensity and segmented per channel by the Otsu’s method [72]. For each species pair we computed the edge-corrected pair-correlation function *g_ij_(r)* = [⟨*a_i_* ⋆ *a_j_*⟩*_r_ / ⟨W ⋆ W⟩_r_*] / (ρ*_i_* ρ*_j_*) from the binary masks *a_i_* using fast Fourier transforms, where *W* is the biomass footprint, ρ_i_ and ρ_j_ are the mean densities of species *i* and *j*, respectively, and ⋆ donates the spatial cross-correlation; *g(r)* = 1 denotes spatially random arrangement; *g(r)* > 1 indicates co-occurrence of two species more than expected (aggregation) and *g(r)* < 1 indicates mutual avoidance (segregation) [73–75]. All analyses were performed in R (version 4.6).

### CAZymes annotation

CAZymes domains were identified by dbCAN3 [76] based on the reference genomes available online (Table S1). Only gene hits that were predicted in at least two of the three tools were selected for the downstream analysis. E value cut-offs for CAZyme categories were optimized on the basis of results matching with the CAZyme database [76].

### Genome-scale metabolic modeling

Metabolic models of the four species used in this study were reconstructed and auto-curated using the gapseq pipeline with default parameters [77]. Manual curation was performed by removing duplicate reactions and metabolites with different IDs, filling network gaps, and adding missing metabolites and reactions. Model quality was assessed by the MEMOTE test suite (version 0.13.0) [78]. The likely exchanged metabolites among the community members and metabolic resource overlaps (MRO) were evaluated using Species METabolic interaction ANAlysis (SMETANA) pipeline [79].

## Results

### Screening carbon sources for synergistic biofilm formation of the SynCom

We used a four-species SynCom, consisting of *Stenotrophomonas rhizophila* (S), *Stenotrophomonas maltophilia* (X), *Microbacterium oxydans* (M), and *Paenibacillus amylolyticus* (P) in this study (Table S1). These bacteria were originally isolated from leaf litter in agricultural soil [64] and have demonstrated synergistic biofilm formation in rich media such as tryptic soy broth (TSB) in our previous work [66, 71, 80]. To assess the influence of different carbon sources on synergistic biofilm formation, the SynCom and monocultures were cultivated in minimal M9 medium supplemented with defined carbon nutrients (Table S2), representing common leaf-derived carbohydrate compounds [81] that range from diffusible sugars (e.g. glucose, cellobiose, xylose) to polymeric long-chain polysaccharides (e.g. cellulose, xylan). CMC was tested as an alternative to natural cellulose due to its solubility in aqueous media [82]. Alginate was also included in the screening, as it has been widely used in 3D printing as a promising matrix material [67].

Across all tested carbon sources, the SynCom consistently produced significantly more biofilm biomass than any monocultures, as quantified by Crystal Violet (CV) staining (Fig. 1B). The robust phenotype of synergistic biofilm formation under diverse nutritional conditions suggested non-additive interspecies interactions, which cannot be simply extrapolated by the sum of each community member (Fig. S1) and showed consistency with our previous studies on the SynCom [8, 71, 83]. Among diffusible substrates, cellobiose supported higher total biofilm biomass than glucose or xylose. Although CMC alone led to minimal biofilm formation, its combination with glucose substantially enhanced biofilm biomass beyond that observed with only glucose, indicating that polymeric substrates can enhance synergistic biofilm development when combined with readily accessible labile carbon sources. Considering these biofilms were submerged in liquid media, we also compared growth curves, which revealed differential planktonic growth dynamics, and surface-associated biomass accumulation across carbon sources (Fig. S2, 1B). For instance, *P. amylolyticus* grew well in both xylose and xylan media, although exhibiting little biofilm formation ability when growing alone. These two readouts capture distinct physiological parameters: growth curves based on planktonic OD reflect metabolic capability under resource-accessible conditions, whereas biofilm biomass stained by the CV assay reflects the emergent consequence of attachment, spatial constraints, and diffusion limitations during biofilm growth in spatially structured environments. We further characterized the influence of carbon source concentrations on biofilm formation (Fig. S3). The SynCom exhibited a strong increase in biofilm biomass production from 1 mM to 10 mM for mono- and di-saccharides of glucose, xylose, and cellobiose, however, the differences were less obvious for polymeric xylan over the range of tested concentrations (0.25–1 wt%), and for mono-species (except for *P. amylolyticus* with cellobiose). These data demonstrated that both the presence and concentration of different carbon sources can influence biofilm development.

We used the CR assay to further reveal carbon resource-dependent differences in extracellular matrix production [68]. CR binds specifically to amyloid-like fibers and certain exopolysaccharides within the biofilm extracellular matrix [69] and resulted in color changes that were recorded by a digital camera (Fig. S4) and analyzed by image processing (Fig. 1C). We observed more intense staining, indicative of altered extracellular matrix production, with dual-, triple-, and quadruple-species consortia comprising *P. amylolyticus,* as compared to mono- or co-cultured consortia without *P. amylolyticus*. This indicates a potential dominant role of *P. amylolyticus* in the SynCom extracellular matrix production (Fig. 1C). A weak color change was noticed with biofilm consortia containing *P. amylolyticus* on the cellulose plates (Fig. 1C, S4), however, all degradation tests using cellulose filter paper showed negative results with monocultures or the SynCom (Fig. S5). Together, these results showed that the type of carbon substrates can modulate multispecies biofilm formation, motivating our further investigation into how these variances translate into spatial organization within biofilms under specific conditions.

### Formulation and 3D printing of chemically defined artificial leaves

The screening experiments demonstrated that the SynCom forms biofilm in a non-additive manner across a range of carbon sources. However, these bulk measurements did not capture how the physical type of resources influences spatial organization within multispecies biofilms. To address this limitation, we next developed a scaffold-based system in which architectural features were constant and different carbon substrates were added in defined physical forms. Because natural leaf litter comprises a heterogeneous mixture of diffusible and polymeric compounds such as simple sugars, cellulose, hemicellulose, lignin, and inorganic minerals [84], it served as an ecological inspiration for the formulation and 3D printing of chemically defined artificial leaves. To reduce the chemical complexity inherent to natural plant residues, while preserving ecological relevance, we fabricated artificial leaf substrates supplemented with defined carbon sources using extrusion-based 3D printing technology (Fig. 2A). Cellulose nanofiber (CNF)-based hydrogel was selected as the matrix of printing materials due to its mechanical stability, optical transparency, and compatibility with downstream cell culturing and imaging [85–87]. All artificial leaves would share the same CNF backbone, ensuring comparable porosity, diffusion properties, and physical micro-structure across the tested conditions as discussed below.

**Figure 2:**
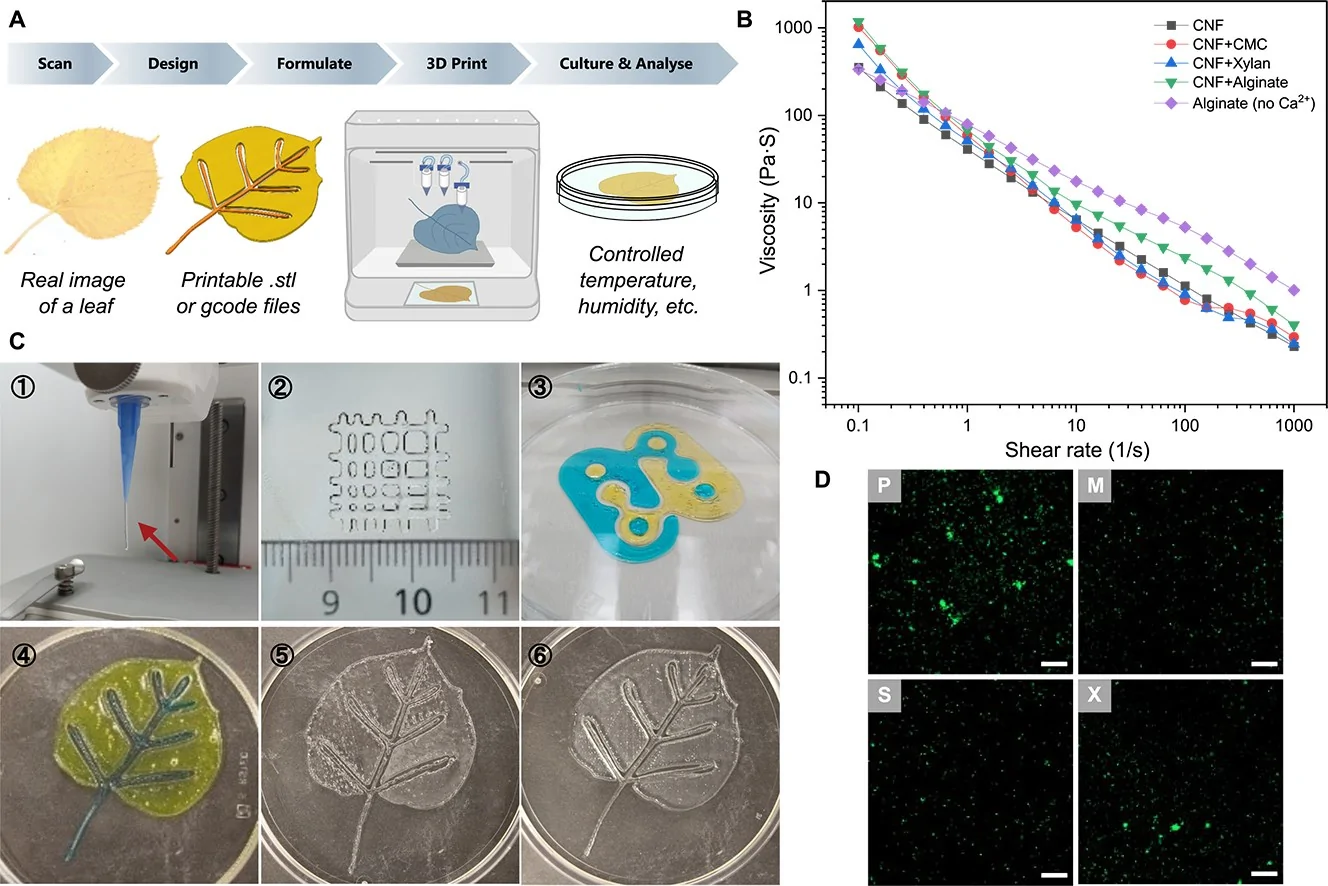
(A) Workflow of 3D printing artificial leaves, including the generation of 3D models by scanning, conversion of the models into printable files, formulation of printable materials, 3D printing, microbial cultivation, and characterization. (B) Rheology property measurements of the formulated hydrogel-based printing materials. CNF: Cellulose nanofiber-based hydrogel. CMC: Carboxymethyl cellulose. (C) Photographs showing an extruded hydrogel filament from the nozzle (1), a printed grid structure with the smallest square edge of 500 μm (2), a printed dual-color logo of our section (3), a printed dual-color artificial leaf (4), and printed artificial leaves made of varied printing materials of microcrystalline cellulose-based hydrogels (5) and CNF hydrogels (6). (D) Fluorescent images showing the living cells of *S. rhizophila* (S), *S. maltophilia* (X), *M. oxydans* (M), and *P. amylolyticus* (P) by staining with SYTO 9 on the artificial leaf made of the CNF hydrogel alone. Scale bar: 20 μm.

Carbon sources were incorporated into the CNF hydrogel formulation prior to printing (Table S3), enabling precise control over chemical composition without imposing spatial patterning of nutrients in the substrates. Rheological characterization confirmed that the addition of diffusible molecules (eg, glucose, cellobiose) or polysaccharides (eg, CMC, xylan) caused only minor changes in printability (Fig. 2B, S6). Although alginate-Ca^2+^ hydrogels exhibited similar good printability (Fig. 2B), we observed precipitation (eg, Ca_3_(PO_4_)_2_) and degradation of the hydrogel structure when incubated in M9 medium, making the alginate-based hydrogel fragile and not suitable for the subsequent experiments. Atomic force microscopy (AFM) showed that the prepared CNF hydrogel comprised 3–4 nm wide and up to 1 μm long nanofibers (Fig. S7). Scanning electron microscopy (SEM) revealed highly interconnected porous networks within the CNF-based substrates with pore sizes exceeding typical bacterial dimensions (Fig. S8), supporting free diffusion of solutes.

Uniform CNF-hydrogel filaments were extruded using the 3D bioprinter (Fig. 2C-1), and the good printability was confirmed by printing a hydrogel-made grid with square edges down to 500 μm (Fig. 2C-2). The ability of printing structured shapes was further demonstrated by formulating the CNF hydrogel with different dye solutions and using two extrusion print heads to construct the logo of our research section (Fig. 2C-3) and an artificial leaf (Fig. 2C-4). Compared to hydrogels made of microcrystalline cellulose (Fig. 2C-5), the printed CNF hydrogel yielded smoother edges, more mechanically robust constructs, and better reproducibility of printing (Fig. 2C-6). Mono-culture experiments confirmed that the printed CNF-hydrogel substrates were biocompatible, leaving a few island-like living cell aggregates (stained with SYTO 9) on top when only supplying the minimum M9 medium without any carbon sources (Fig. 2D). No detectable background signal or dye residue was introduced by the CNF-hydrogel substrates during fluorescence microscopy imaging. These properties allowed us to examine how different carbon source formats, rather than the physical structure, shape biofilm assembly on these 3D-printed, reproducible substrates.

### Carbon source type drives distinct spatial self-organization of multispecies biofilms

To investigate the role of carbon source type on biofilm assembly, instead of predefining the spatial organization of microbial cells embedded in hydrogel matrix as done in earlier studies [51–54], we incorporated defined carbon sources in CNF hydrogels prior to printing and inoculated microbial cultures on top of the substrates to allow biofilms to grow and mature naturally (Fig. 3A). Microscopic imaging by confocal laser scanning microscopy (CLSM) revealed that the spatial organization of SynCom biofilms strongly depended on the carbon source composition (Fig. 3B). CNF and CNF + CMC yielded similar total biofilm volume and average biofilm thickness (Fig. 3C); however, small and sparse aggregates were formed in the presence of only CNF, whereas bigger and close-packed biofilms were developed when CMC was present (Fig. 3B). Moreover, the addition of xylan supported more extensive and substantially thicker biofilms, as compared to biofilm growth with CNF only (Fig. 3B and 3C). Live/dead cell staining results showed comparable overall viability across the tested substrates but indicated uneven distribution of dead cells particularly with xylan (Fig. 3B and 3D).

**Figure 3:**
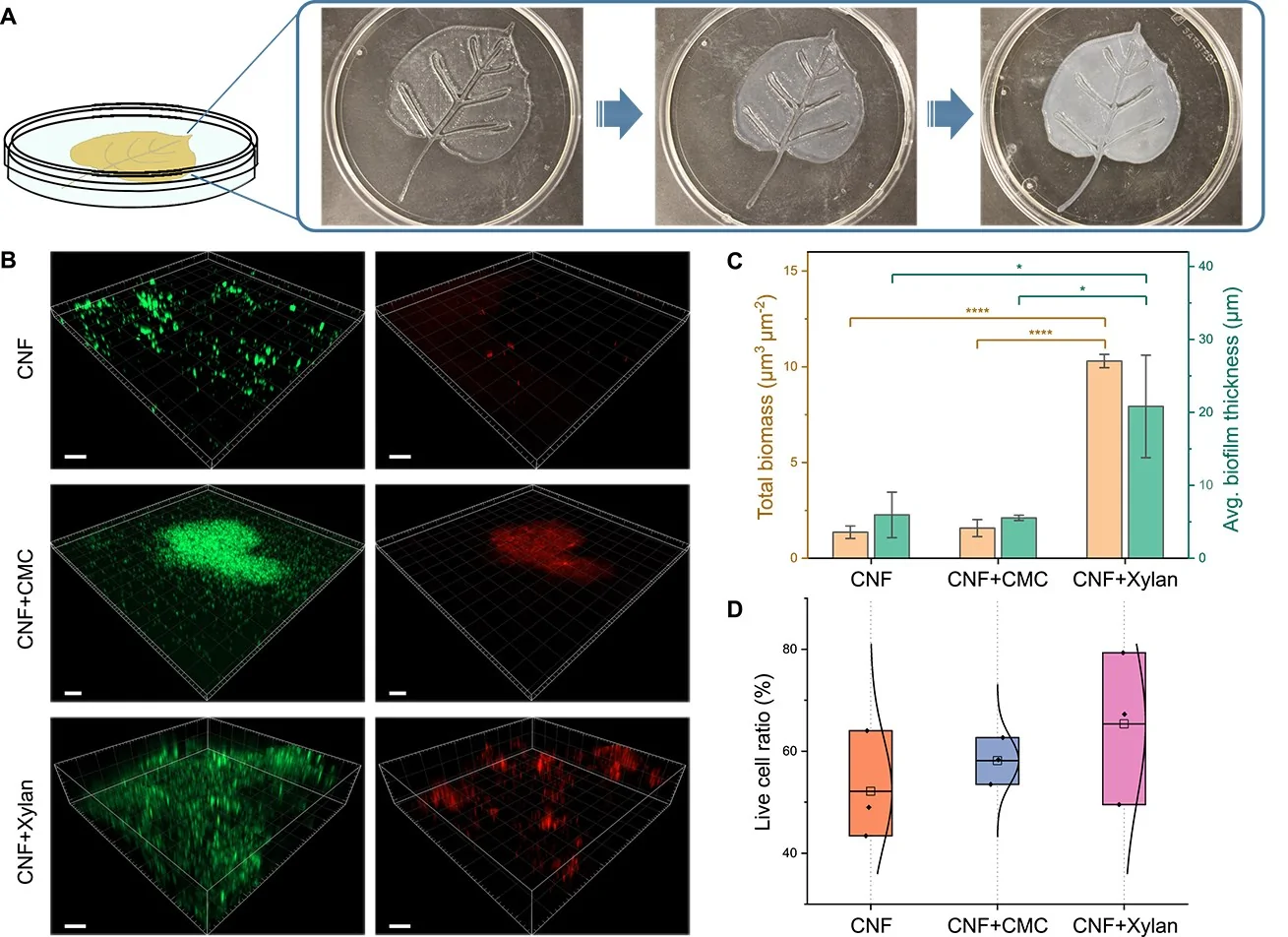
(A) Illustration of the culture system on printed substrates. From left to right: A petri dish container sealed with a gas-permeable but water-proof membrane, and images showing the freshly printed substrate, the substrate after inoculation and with biofilm formed on the top. (B) Fluorescent images showing the live/dead-stained multispecies biofilms formed on the cellulose nanofiber (CNF)-based substrates containing different carbon sources. CNF + CMC: CNF with carboxymethyl cellulose, CNF + Xylan: CNF with Xylan. The left panels show live cells stained with SYTO 9, whereas the right panels show dead cells stained with propidium iodide (PI). Scale bar: 20 μm. (C) Quantification of total biofilm biomass and averaged biofilm thickness. Statistical significance is shown as ^*^  *P* < 0.05, ^**^  *P* < 0.01, ^***^  *P* < 0.001, ^****^  *P* < 0.0001 (one-sided Welch’s *t-*test). Error bars show standard deviation (*n* = 3). (D) The living cell ratio within each biofilm formed on the substrates with different carbon sources of CNF, CNF + CMC, and CNF + Xylan.

To address the core question of “who is where”, we applied specific oligonucleotide probes labeled with unique fluorochromes (Table S4) and used FISH to enable analysis of species-level localization within multispecies biofilms [88]. On CNF-only substrates, biofilm coverage was limited and dominated by *P. amylolyticus* (Fig. 4A). The addition of cellobiose, an intermediate during cellulose degradation [89] that supported the growth of *P. amylolyticus* (Fig. S2B), increased total biofilm coverage and maintained the coexistence of multiple species, including *S. rhizophila*, which did not form substantial biofilms on cellobiose alone (Figs 1B and 4B). In contrast, substrates containing both cellobiose and polymeric carbon sources (CMC or xylan) promoted highly structured biofilms, in which *P. amylolyticus* occupied peripheral regions surrounding other community members (Fig. 4C and 4D). Local fraction and pair correlation analysis of the FISH images (Fig. 4E and 4F) showed that the four strains were randomly intermixed on the cellobiose-alone supplement at all scales (*g(r)* ≈ 1), whereas the polymer-supplemented communities were strongly structured with M/S/X co-aggregated into a core, from which *P. amylolyticus* was excluded at short range and became enriched at the periphery (Fig. S9). These observations suggest that the type of carbon substrate strongly regulates spatial organization within multispecies biofilms, where the addition of polymeric, less labile substrates on top of a basal cellobiose supply leads to structured species distribution characterized by localized dominant species.

**Figure 4:**
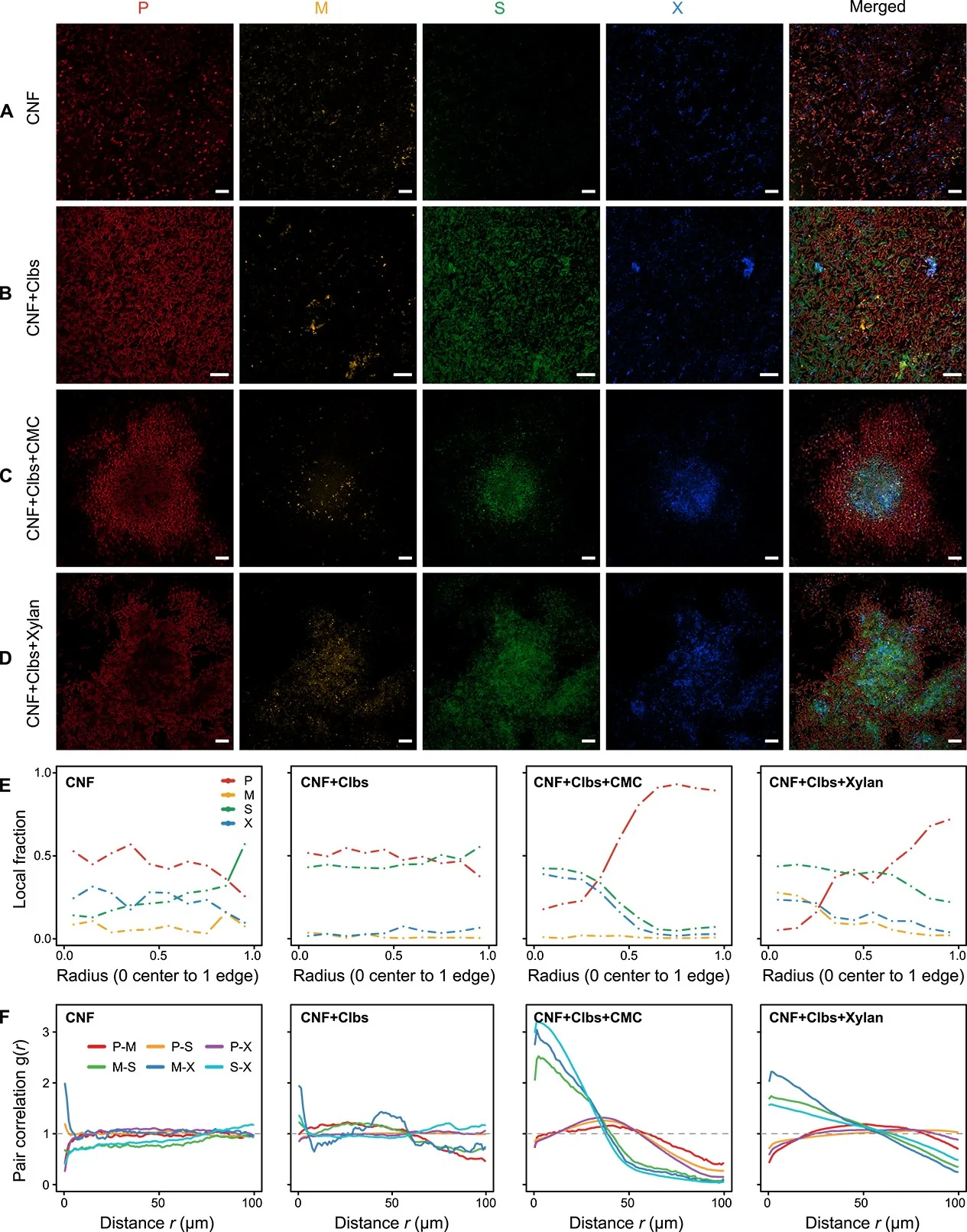
(A-D) Single-channel and merged confocal images showing the spatial structures of the four-species SynCom biofilms on different carbon sources of panel (A) cellulose nanofiber (CNF), (B) CNF with cellobiose (CNF + Clbs), (C) CNF with cellobiose and carboxymethyl cellulose (CNF + Clbs+CMC), and (D) CNF with cellobiose and Xylan (CNF + Clbs+Xylan). Columns show species-specific fluorescence *in situ* hybridization signals for *P. amylolyticus* (P), *M. oxydans* (M), *S. rhizophila* (S), and *S. maltophilia* (X), followed by the merged images. Scale bar: 20 μm. (E) Local fraction of each species versus normalized radius from the microcolony centroid (0 = core, 1 = edge). (F) Pair-correlation functions *g(r)* for all six species pairs versus inter-cell distance *r*: *g(r)* = 1 (grey dotted line) denotes a spatially random arrangement, *g(r)* > 1 co-aggregation and *g(r)* < 1 segregation. *g(r)* was computed from Otsu-segmented channels by FFT-based pair correlation.

### Genome-informed prediction of metabolic interaction potential in the SynCom

To further explore the metabolic capacities and potential interactions in the SynCom, we annotated the genomes of each strain against the CAZyme database (dbCAN3) [76] and revealed substantial differences in CAZyme repertories among SynCom members (Fig. 5A). *P. amylolyticus* encoded an almost three-fold higher number of gene hits covering all six CAZyme families, as compared to the other three species. This includes enzymes implicated in the degradation of cellulose (e.g. endoglucanase (EC 3.2.1.4), exoglucanase (EC 3.2.1.91), β-glucosidase (EC 3.2.1.21)) and xylan (e.g. endo-1,4-β-xylanase (EC 3.2.1.8), acetylxylan esterase (EC 3.2.1.72), arabinofuranosidase (EC 3.2.1.55), β-1,4-xylosidase (EC 3.2.1.37)) (Fig. 5B), and is consistent with *P. amylolyticus* playing an apparent dominant role on polymer-containing substrates [90]. The other three species possessed limited repertoires of CAZymes, indicating that their ability to exploit more complex substrates is limited. However, the filter paper cellulose degradation assay (Fig. S4) provided partial negative experimental evidence for crystalline cellulose hydrolysis, as well as the use of more soluble CMC as a cellulose substitute in the CV assays (Fig. 1B). Future work using zymography under optimized culture conditions is needed for direct validation of predicted CAZyme functions.

**Figure 5:**
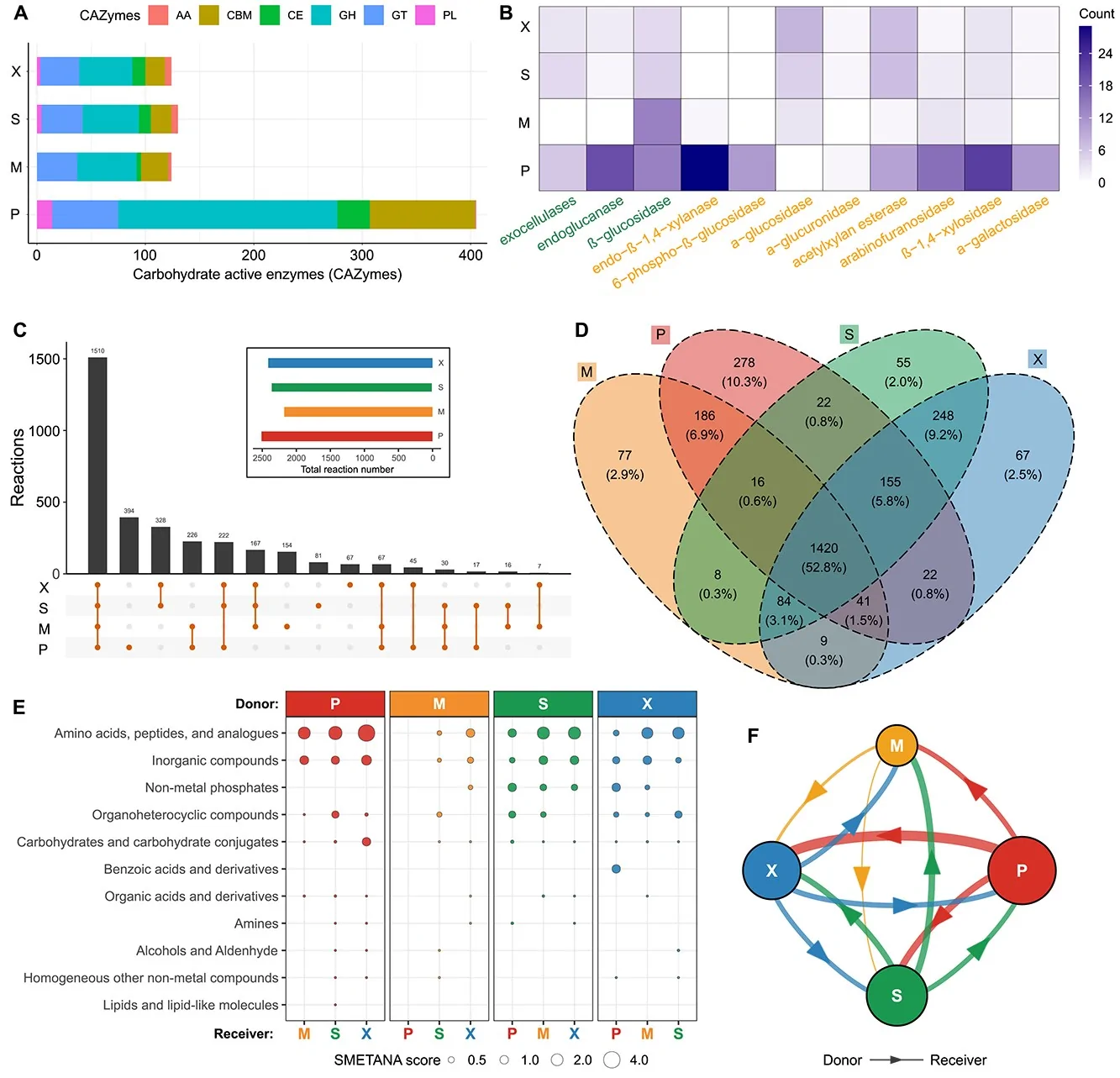
(A) Annotation of carbohydrate-active enZymes (CAZymes) including auxiliary activity (AA), glycoside hydrolase (GH), glycosyl transferase (GT), carbohydrate esterase (CE), polysaccharide lyase (PL) families and carbohydrate-binding modules (CBM) of the four species (P: *P. amylolyticus*, M: *M. oxydans*, S: *S. rhizophila*, X: *S. maltophilia*). (B) Annotated genes predicted to contribute to cellulose and xylan degradation, identified by the corresponding substrate labels. (C) The overlap of all reactions predicted by the genome-scale metabolic modeling of the SynCom members. (D) The overlap of all metabolites predicted by the genome-scale metabolic modeling of the SynCom members. (E) Potential metabolic exchanges among the SynCom members predicted using SMETANA. Bubble size is proportional to the SMETANA score. (F) Network of predicted metabolic interactions. Arrows point from donor to receiver, and line thickness is proportional to the SMETANA score.

We reconstructed genome-scale metabolic models (GEMs) by using the whole-genome sequences of each SynCom member [91]. GEMs are mathematical reconstructions of all known metabolic reactions encoded in an organism’s genome. The predictive models showed that 46.3% of the reactions (Fig. 5C) and 52.8% of the metabolites (Fig. 5D) overlapped among the four strains composing the SynCom. The two Gram-positive strains, *P. amylolyticus* and *M. oxydans,* contained more unique metabolic reactions and metabolites related to biochemical pathways involving amino acids and derivatives, carbohydrates, nitrogen and sulfur metabolism, and membrane transportation. In contrast, more distinct metabolic processes involving fatty acids, lipids, and oxidative stress response were uncovered in the two Gram-negative strains, *S. maltophilia* and *S. rhizophila*. This observation supports the findings from our previous study using the same SynCom, showing accelerated keratin degradation rates and an increased abundance of proteins (e.g. glutathione peroxidase) related to oxidative stress in *S. maltophilia* when co-cultured with other community members [92]. Emergent community-properties may thus arise due to interspecies interactions via activation or deactivation of specific pathways in response to environmental conditions.

GEMs-based analysis by SMETANA scores, which represent the combined probability and necessity of a specific metabolite transfer between two species, further predicted extensive metabolic interactions within the SynCom, with *P. amylolyticus* acting as a major donor of potential metabolic exchanges (Fig. 5E, Table S5). The top-scored metabolites for such inter-species exchange included amino acids, inorganic compounds, carbohydrates, and organic acids. Our analysis indicates the presence of potentially a more complex metabolic interaction network in the SynCom (Fig. 5F). Consistent with these predicted bidirectional exchanges, every SynCom member including *P. amylolyticus* formed more biofilm in the community-conditioned supernatants (PMSX-sup) than the *P. amylolyticus*-conditioned supernatants (P-sup) across most tested substrates (Fig. S10). This pattern may suggest reciprocal cross-feeding rather than a one-way degrader-exploiter-scavenger relationship (Fig. 5F, Fig. S10). This prediction also aligns with previous metatranscriptomics analyses of the SynCom, showing that differentially expressed genes were highly affiliated to the metabolic processes involving amino acid biosynthesis, membrane transport, and energy conversion [93].

A high MRO (0.79, calculated according to Zelezniak et al. [79]) among the SynCom members suggested substantial competition for shared resources, particularly in the absence of *P. amylolyticus*, which resulted in an even higher MRO of 0.87 indicative of greater potential for resource competition. Together, the genomic annotation and metabolic modeling provide a constrained yet informative picture of the metabolic interaction potential within the SynCom. Although these analyses do not directly resolve realized interactions or spatial organization, the predictions suggest that resource-driven metabolic asymmetries may bias how microbial interactions are expressed in space. However, these predictions remain bioinformatic inferences and have not been directly validated e.g. by metabolomics measurements. Elucidating how such metabolic potentials are translated into emergent spatial and functional organization will require future integration of spatially explicit modeling frameworks [4] and quantitative metabolite measurements [94].

## Discussion

Despite substantial advances driven by high-throughput sequencing and system-level analyses, general principles governing the assembly of microbial communities particularly in biofilms remain elusive [95]. Biofilms represent the dominant microbial lifestyle in many natural and engineered ecosystems, yet their intrinsic spatial heterogeneity complicates the interpretation of species interactions and community functions [9, 12, 96–98]. In this study, we examined how the type of carbon substrate shapes the assembly, spatial organization, and inferred metabolic interactions of a four-species SynCom. Using 3D-printed, CNF-based substrates that mimic aspects of leaf litter degradation, we demonstrated that polymeric carbon sources such as CMC (carboxymethyl group-modified cellulose from plants) and xylan (one type of hemicellulose) profoundly alter biofilm architecture, as compared to biofilm development with more labile and diffusible carbon sources such as cellobiose. Even though synergistic biofilm formation and viability remained comparable across the tested conditions (currently limited at a single developmental time-point measurement), the spatial distribution of cells and species differed markedly, indicating an important role of carbon substrate type in modulating the spatial organization of biofilm community members. The type of carbon substrate thus appears as an important ecological variable, which can generate spatial niches in chemically defined and physically consistent environments.

The formation of aggregated and spatially partitioned biofilm structures on polymer-containing substrates likely reflects localized access to degradation products released via extracellular enzymatic activity [9, 11, 99–101]. In contrast to freely diffusible substrates, polymeric carbon sources require enzymatic depolymerization, effectively constraining carbon availability to regions near active degraders [9, 42]. Such constraints can promote spatial segregation and structured biofilm growth, consistent with ecological theory predicting that diffusion limitation and localized resource conversion foster spatial patterning in microbial communities [102–104]. Such spatial effects arose in our study despite the use of a consistent hydrogel backbone across all printed constructs, indicating that differences in biofilm architecture were driven primarily by chemical resource type rather than physical scaffold heterogeneity. This distinction is critical, as it suggests that even subtle changes in resource chemistry can lead to a spatial reorganization of microbial biofilm communities without altering bulk material properties.

Previous applications of 3D printing in biofilm studies have largely focused on embedding cells or defining their initial spatial arrangements [51–54]. In contrast, our study used 3D printing to standardize the physical environment, while selectively varying chemical resource composition, allowing biofilm structure to emerge naturally through microbial growth and interaction. A limitation of our current artificial leaf design is that it captures only a subset of the chemical and structural complexity of natural leaf litter. The extrusion-based 3D bioprinter used in this study achieves a minimum feature size of ~500 μm, as demonstrated by the grid structure in Fig. 2C, and does not faithfully reproduce sub-micron leaf surface features or fine-scale topography such as cuticular wax crystallites or stomatal openings [105–107]. Even though extrusion-based 3D printing offers opportunities to incorporate additional polymers or gradients in future designs, the present system should be viewed as a reductionist, yet tractable platform for dissecting how defined environmental variables influence multispecies biofilms and supporting continuous biofilm development monitoring.

Genome annotation and metabolic modeling revealed substantial differences in the CAZymes repertoires among the SynCom members, with *P. amylolyticus* encoding a disproportionately large capacity for polymer degradation. This metabolic potential of *P. amylolyticus* aligned with its dominant spatial positioning in biofilms grown on polymer-containing substrates, where it surrounded or overlaid other species. However, this dominance was not universal across conditions, emphasizing that functional complexity emerges from the interaction between metabolic capacity and environmental context, rather than being an intrinsic property of individual species [104, 108, 109]. GEM further predicted extensive metabolic overlap among community members, suggesting strong potential for competition. Despite this predicted metabolic overlap, the consortium consistently exhibited synergistic biofilm formation across tested conditions. This suggests that interaction outcomes in structured biofilms cannot be inferred from metabolic network overlap alone, and that spatial organization and localized substrate accessibility may modulate how competitive and potentially synergistic interactions are realized. These findings underscore the importance of considering spatial context when interpreting metabolic interaction networks and community-level phenotypes [110–112].

However, we also note the need for further improvements of our current framework for metabolic modeling analysis [4, 113–115]. Although the predicted role of *P. amylolyticus* as a major donor of metabolites, coupled with high metabolic resource overlaps, suggests that both cooperation and competition coexist within the community, these predictions remain inherently non-spatial and therefore cannot fully capture how metabolite exchange is realized within structured biofilms. Rather than claiming direct causality between predicted metabolic exchanges and observed spatial patterns, our results support a more nuanced interpretation that carbon substrate type shapes spatial organization, which in turn modulates the expression and consequences of metabolic interaction potential. Resolving how these processes dynamically reinforce one another requires future integration of spatially explicit modeling (e.g. BacArena [116], COMETS [117]) with quantitative metabolite measurements (e.g. stable isotope probing and imaging [118]), and time-resolved analyses (e.g. labeling and spectral imaging [119]) for experimental validation.

Overall, our findings suggest that resource-imposed spatial constraints may be a fundamental driver of microbial community organization, cooperation, and function during multispecies biofilm assembly. Our study highlights the necessity of integrating carbon substrate type, spatial organization, and metabolic potential to understand multispecies biofilm ecology. By combining controlled experimental systems with genome-informed modeling, we provide a framework for probing how environmental factors govern microbial community assembly. Further development of spatially resolved experimental and computational tools will be essential for translating these insights into predictive models of biofilm behavior in natural and engineered ecosystems.

## Supplementary Material

Supplementary_material_wrag223

## Data and materials availability

The genome sequences of the four strains used in this study are available from the European Nucleotide Archive (ENA) by accession numbers PRJEB15263, PRJEB18431, PRJEB15265 and PRJEB15262. All experimental data and code are available on Zenodo (DOI: 10.5281/zenodo.21278269).

## References

[1] Flemming HC, Wingender J, Szewzyk U., et al. Biofilms: an emergent form of bacterial life. *Nat Rev Microbiol* 2016;14:563–75. 10.1038/nrmicro.2016.94 27510863

[2] Liu W, Røder HL, Madsen JS., et al. Interspecific bacterial interactions are reflected in multispecies biofilm spatial organization. *Front Microbiol* 2016;7:1366. 10.3389/fmicb.2016.01366 PMC5005372 27630624

[3] Madsen JS, Sørensen SJ, Burmølle M. Bacterial social interactions and the emergence of community-intrinsic properties. *Curr Opin Microbiol* 2018;42:104–9. 10.1016/j.mib.2017.11.018 29197823

[4] van den Berg NI, Machado D, Santos S., et al. Ecological modelling approaches for predicting emergent properties in microbial communities. *Nat Ecol Evol* 2022;6:855–65. 10.1038/s41559-022-01746-7 PMC7613029 35577982

[5] Squyres GR, Newman DK. Biofilms as more than the sum of their parts: lessons from developmental biology. *Curr Opin Microbiol* 2024;82:102537. 10.1016/j.mib.2024.102537 PMC12124288 39241276

[6] Wang LJ, Wang X, Wu H., et al. Interspecies synergistic interactions mediated by cofactor exchange enhance stress tolerance by inducing biofilm formation. *Msystems* 2024;9:e0088424–4. 10.1128/msystems.00884-24 PMC11406921 39189769

[7] Rendueles O, Ghigo JM. Mechanisms of competition in biofilm communities. *Microbiol Spectrum* 2015;3:319–42. 10.1128/microbiolspec.MB-0009-2014 26185066

[8] Ren D, Madsen JS, Sørensen SJ., et al. High prevalence of biofilm synergy among bacterial soil isolates in cocultures indicates bacterial interspecific cooperation. *ISME J.* 2015;9:81–9. 10.1038/ismej.2014.96 PMC4274433 24936766

[9] Jo JY, Price-Whelan A, Dietrich LEP. Gradients and consequences of heterogeneity in biofilms. *Nat Rev Microbiol* 2022;20:593–607. 10.1038/s41579-022-00692-2 PMC9590228 35149841

[10] Valiei A, Dickson A, Aminian-Dehkordi J., et al. Metabolic interactions shape emergent biofilm structures in a conceptual model of gut mucosal bacterial communities. *Npj Biofilms Microbi* 2024;10:99. 10.1038/s41522-024-00572-y PMC11447261 39358363

[11] Flemming HC, van Hullebusch ED, Neu TR., et al. The biofilm matrix: multitasking in a shared space. *Nat Rev Microbiol.* 2023;21:70–86. 10.1038/s41579-022-00791-0 36127518

[12] Rumbaugh KP, Whiteley M. Towards improved biofilm models. *Nat Rev Microbiol.* 2025;23:57–66. 10.1038/s41579-024-01086-2 39112554

[13] Karygianni L, Ren Z, Koo H., et al. Biofilm matrixome: extracellular components in structured microbial communities. *Trends Microbiol* 2020;28:668–81. 10.1016/j.tim.2020.03.016 32663461

[14] Bridier A, Briandet R. Microbial biofilms: structural plasticity and emerging properties. *Microorganisms.* 2022;10:138. 10.3390/microorganisms10010138 PMC8778831 35056587

[15] Roder HL, Sørensen SJ, Burmølle M. Studying bacterial multispecies biofilms: where to start? *Trends Microbiol* 2016;24:503–13. 10.1016/j.tim.2016.02.019 27004827

[16] Elias S, Banin E. Multi-species biofilms: living with friendly neighbors. *FEMS Microbiol Rev* 2012;36:990–1004. 10.1111/j.1574-6976.2012.00325.x 22229800

[17] Joshi RV, Gunawan C, Mann R. We are one: multispecies metabolism of a biofilm consortium and their treatment strategies. *Front Microbiol* 2021;12:635432. 10.3389/fmicb.2021.635432 PMC7876221 33584635

[18] Pierce EC, Dutton RJ. Putting microbial interactions back into community contexts. *Curr Opin Microbiol* 2022;65:56–63. 10.1016/j.mib.2021.10.008 34739927

[19] Eigentler L, Davidson FA, Stanley-Wall NR. Mechanisms driving spatial distribution of residents in colony biofilms: an interdisciplinary perspective. *Open Biol* 2022;12:220194. 10.1098/rsob.220194 PMC9748781 36514980

[20] Cremin K, Duxbury SJN, Rosko J., et al. Formation and emergent dynamics of spatially organized microbial systems. *Interface Focus* 2023;13:20220062. 10.1098/rsfs.2022.0062 PMC9912014 36789239

[21] Lou YY, Liu Z, Zhang Q., et al. Interspecies and intraspecies 'talk' shape the bacterial biofilms. *Food Qual Saf-Oxford* 2025;9:fyaf008. 10.1093/fqsafe/fyaf008

[22] Wang J, Hashem I, Bhonsale S., et al. Individual-based modeling unravels spatial and social interactions in bacterial communities. *ISME J.* 2025;19:wraf116. 10.1093/ismejo/wraf116 PMC12411854 40838740

[23] Ono H, Tsuru S, Furusawa C. Carbon source diversity shapes bacterial interspecies interactions. *ISME J.* 2025;19:wraf224. 10.1093/ismejo/wraf224 PMC12599325 41060295

[24] Schäfer M, Pacheco AR, Künzler R., et al. Metabolic interaction models recapitulate leaf microbiota ecology. *Science.* 2023;381:eadf5121. 10.1126/science.adf5121 37410834

[25] Dal Bello M, Lee H, Goyal A., et al. Resource-diversity relationships in bacterial communities reflect the network structure of microbial metabolism. *Nat Ecol Evol* 2021;5:1424–34. 10.1038/s41559-021-01535-8 34413507

[26] Wang DY, Hunt KA, Candry P., et al. Cross-feedings, competition, and positive and negative synergies in a four-species synthetic community for anaerobic degradation of cellulose to methane. *Mbio.* 2023;14:e03189–22. 10.1128/mbio.03189-22 PMC10128006 36847519

[27] Dey S, Nayak AK, Dhiman R., et al. Pleomorphism drives the lifestyle transitions in bacteria for micro-niche adaptation in biofilm. *Rev Environ Sci Bio* 2025;24:831–60. 10.1007/s11157-025-09742-6

[28] Chacón JM, Möbius W, Harcombe WR. The spatial and metabolic basis of colony size variation. *ISME J.* 2018;12:669–80. 10.1038/s41396-017-0038-0 PMC5864198 29367665

[29] Hall-Stoodley L, Costerton JW, Stoodley P. Bacterial biofilms: from the natural environment to infectious diseases. *Nat Rev Microbiol* 2004;2:95–108. 10.1038/nrmicro821 15040259

[30] Rajewska M, Maciąg T, Narajczyk M., et al. Carbon source and substrate surface affect biofilm formation by the plant-associated bacterium pseudomonas donghuensis p482. *Int J Mol Sci* 2024;25:8351. 10.3390/ijms25158351 PMC11312691 39125921

[31] Scheidweiler D, Bordoloi AD, Jiao W., et al. Spatial structure, chemotaxis and quorum sensing shape bacterial biomass accumulation in complex porous media. *Nat Commun* 2024;15:191. 10.1038/s41467-023-44267-y PMC10761857 38167276

[32] Yang XR, Yang T, Zhang Z., et al. Substrate utilization and cross-feeding synergistically determine microbiome resistance to pathogen invasion. *Nat Ecol Evol* 2025;10:211–20. 10.1038/s41559-025-02908-z 41272314

[33] Gu Y, Yan W, Chen Y., et al. Plant growth-promotion triggered by extracellular polymer is associated with facilitation of bacterial cross-feeding networks of the rhizosphere. *ISME J* 2025;19:wrag040. 10.1093/ismejo/wraf040 PMC11937826 40037574

[34] Jans M, Sarremejane R, del Campo R., et al. Changes in leaf-litter chemistry and microbial communities drive leaf-litter decomposition across river terrestrial-aquatic habitats. *Freshw Biol* 2025;70:e70074. 10.1111/fwb.70074

[35] Candry P, Godfrey BJ, Winkler MKH. Microbe-cellulose hydrogels as a model system for particulate carbon degradation in soil aggregates. *Isme Commun* 2024;4:ycae068. 10.1093/ismeco/ycae068 PMC11126157 38800124

[36] Stubbusch AKM, Keegstra JM, Schwartzman J., et al. Polysaccharide breakdown products drive degradation-dispersal cycles of foraging bacteria through changes in metabolism and motility. *Elife.* 2024;13:RP93855. 10.7554/eLife.93855 PMC11493405 39429128

[37] Stubbusch AKM, Peaudecerf FJ, Lee KS., et al. Antagonism as a foraging strategy in microbial communities. *Science.* 2025;388:1214–7. 10.1126/science.adr8286 40504916

[38] D'Souza G, Schwartzman J, Keegstra J., et al. Interspecies interactions determine growth dynamics of biopolymer-degrading populations in microbial communities. *PNAS.* 2023;120:e2305198120. 10.1073/pnas.2305198120 PMC10622921 37878716

[39] Kommedal EG, Angeltveit CF, Klau LJ., et al. Visible light-exposed lignin facilitates cellulose solubilization by lytic polysaccharide monooxygenases. *Nat Commun* 2023;14:1063. 10.1038/s41467-023-36660-4 PMC9958194 36828821

[40] Sichert A, Cordero OX. Polysaccharide-bacteria interactions from the lens of evolutionary ecology. *Front Microbiol* 2021;12:705082. 10.3389/fmicb.2021.705082 PMC8531407 34690949

[41] Bistarelli LT, Poyntner C, Santín C., et al. Wildfire-derived pyrogenic carbon modulates riverine organic matter and biofilm enzyme activities in an in situ flume experiment. *ACS EST Water* 2021;1:1648–56. 10.1021/acsestwater.1c00185 PMC8276270 34278381

[42] Wang ZW, Lee SH, Elkins JG., et al. Spatial and temporal dynamics of cellulose degradation and biofilm formation by caldicellulosiruptor obsidiansis and clostridium thermocellum. *AMB Express* 2011;1:30. 10.1186/2191-0855-1-30 PMC3222322 21982458

[43] Ziemba C, Shabtai Y, Piatkovsky M., et al. Cellulose effects on morphology and elasticity of vibrio fischeri biofilms. *Npj Biofilms Microbi* 2016;2:1. 10.1038/s41522-016-0001-2 PMC5460256 28649395

[44] Banitz T, Wick LY, Fetzer I., et al. Dispersal networks for enhancing bacterial degradation in heterogeneous environments. *Environ Pollut* 2011;159:2781–8. 10.1016/j.envpol.2011.05.008 21645953

[45] Depetris A, Tagliavini G, Peter H., et al. Biophysical properties at patch scale shape the metabolism of biofilm landscapes. *Npj Biofilms Microbi.* 2022;8:5. 10.1038/s41522-022-00269-0 PMC8813951 35115555

[46] Pontrelli S, Szabo R, Pollak S., et al. Metabolic cross-feeding structures the assembly of polysaccharide degrading communities. Science. *Advances.* 2022;8:eabk3076. 10.1126/sciadv.abk3076 PMC8865766 35196097

[47] Miao YZ, Wang W, Xu H., et al. A novel decomposer-exploiter interaction framework of plant residue microbial decomposition. *Genome Biol* 2025;26:20. 10.1186/s13059-025-03486-w PMC11792400 39901283

[48] Abdoli P, Vulin C, Lepiz M., et al. Substrate complexity buffers negative interactions in a synthetic community of leaf litter degraders. *FEMS Microbiol Ecol* 2024;100:fiae102. 10.1093/femsec/fiae102 PMC11289631 39020097

[49] Sulheim S, Mitri S. Breaking down microbial hierarchies. *Trends Microbiol* 2023;31:426–7. 10.1016/j.tim.2023.02.014 36935220

[50] Nadell CD, Drescher K, Foster KR. Spatial structure, cooperation and competition in biofilms. *Nat Rev Microbiol.* 2016;14:589–600. 10.1038/nrmicro.2016.84 27452230

[51] Connell JL, Ritschdorff ET, Whiteley M., et al. 3d printing of microscopic bacterial communities. *PNAS.* 2013;110:18380–5. 10.1073/pnas.1309729110 PMC3832025 24101503

[52] Kumar RK, Foster KR. 3d printing of microbial communities: a new platform for understanding and engineering microbiomes. *Microb Biotechnol* 2023;16:489–93. 10.1111/1751-7915.14168 PMC9948180 36511313

[53] Kumar RK, Meiller-Legrand TA, Alcinesio A., et al. Droplet printing reveals the importance of micron-scale structure for bacterial ecology. *Nat Commun* 2021;12:857. 10.1038/s41467-021-20996-w PMC7870943 33558498

[54] Schmieden DT, Basalo Vázquez SJ, Sangüesa H., et al. Printing of patterned, engineered e. coli biofilms with a low-cost 3d printer. *ACS Synth Biol* 2018;7:1328–37. 10.1021/acssynbio.7b00424 29690761

[55] Krujatz F, Dani F, Windisch J., et al. Think outside the box: 3d bioprinting concepts for biotechnological applications - recent developments and future perspectives. *Biotechnol Adv* 2022;58:107930. 10.1016/j.biotechadv.2022.107930 35257786

[56] Lazarus E, Meyer AS, Ikuma K., et al. Three dimensional printed biofilms: fabrication, design and future biomedical and environmental applications. *Microb Biotechnol* 2024;17:e14360. 10.1111/1751-7915.14360 PMC10832517 38041693

[57] Rodrigo-Navarro A, Sankaran S, Dalby MJ., et al. Engineered living biomaterials. *Nat Rev Mater* 2021;6:1175–90. 10.1038/s41578-021-00350-8

[58] Kadilak AL, Rehaag JC, Harrington CA., et al. A 3d-printed microbial cell culture platform with in situ pegda hydrogel barriers for differential substrate delivery. *Biomicrofluidics.* 2017;11:054109. 10.1063/1.5003477 PMC5624803 29034053

[59] Bay RK, Hancock AM, Dill-Macky AS., et al. 3d printing bacteria to study motility and growth in complex 3d porous media. *JoVE Journal* 2024;203:e66166. 10.3791/66166 38314825

[60] Cui ZH, Feng Y, Liu F., et al. 3d bioprinting of living materials for structure-dependent production of hyaluronic acid. *ACS Macro Lett* 2022;11:452–9. 10.1021/acsmacrolett.2c00037 35575323

[61] Koo H, Yamada KM. Dynamic cell-matrix interactions modulate microbial biofilm and tissue 3d microenvironments. *Curr Opin Cell Biol* 2016;42:102–12. 10.1016/j.ceb.2016.05.005 PMC5064909 27257751

[62] Paula AJ, Hwang G, Koo H. Dynamics of bacterial population growth in biofilms resemble spatial and structural aspects of urbanization. *Nat Commun* 2020;11:1354. 10.1038/s41467-020-15165-4 PMC7070081 32170131

[63] Guzmán-Soto I, McTiernan C, Gonzalez-Gomez M., et al. Mimicking biofilm formation and development: recent progress in in vitro and in vivo biofilm models. *Iscience.* 2021;24:102443. 10.1016/j.isci.2021.102443 PMC8113887 34013169

[64] de la Cruz-Perera CI, Ren D, Blanchet M., et al. The ability of soil bacteria to receive the conjugative incp1 plasmid, pkjk10, is different in a mixed community compared to single strains. *FEMS Microbiol Lett* 2013;338:95–100. 10.1111/1574-6968.12036 23106414

[65] Svagan AJ, Bender Koch C, Hedenqvist MS., et al. Liquid-core nanocellulose-shell capsules with tunable oxygen permeability. *Carbohyd Polym* 2016;136:292–9. 10.1016/j.carbpol.2015.09.040 26572358

[66] Ren D, Madsen JS, de la Cruz-Perera CI., et al. High-throughput screening of multispecies biofilm formation and quantitative pcr-based assessment of individual species proportions, useful for exploring interspecific bacterial interactions. *Microb Ecol* 2014;68:146–54. 10.1007/s00248-013-0315-z 24337804

[67] Chivers PRA, Smith DK. Shaping and structuring supramolecular gels. *Nat Rev Mater* 2019;4:463–78. 10.1038/s41578-019-0111-6

[68] Kaiser TDL, Pereira EM, dos Santos KRN., et al. Modification of the Congo red agar method to detect biofilm production by staphylococcus epidermidis. *Diagn Micr Infec Dis* 2013;75:235–9. 10.1016/j.diagmicrobio.2012.11.014 23313084

[69] Jones CJ, Wozniak DJ. Congo red stain identifies matrix overproduction and is an indirect measurement for c-di-gmp in many species of bacteria. *Methods Mol Biol* 2017;1657:147–56. 10.1007/978-1-4939-7240-1_12 28889292

[70] Heydorn A, Nielsen AT, Hentzer M., et al. Quantification of biofilm structures by the novel computer program comstat. *Microbiology.* 2000;146:2395–407. 10.1099/00221287-146-10-2395 11021916

[71] Liu W, Russel J, Burmølle M., et al. Micro-scale intermixing: a requisite for stable and synergistic co-establishment in a four-species biofilm. *ISME J.* 2018;12:1940–51. 10.1038/s41396-018-0112-2 PMC6052071 29670216

[72] Otsu N . A threshold selection method from gray-level histograms. *IEEE Transactions on Systems, Man, and Cybernetics* 1979;9:62–6. 10.1109/TSMC.1979.4310076

[73] Sengupta P, Jovanovic-Talisman T, Skoko D., et al. Probing protein heterogeneity in the plasma membrane using palm and pair correlation analysis. *Nat Methods* 2011;8:969–75. 10.1038/Nmeth.1704 PMC3400087 21926998

[74] Veatch SL, Machta BB, Shelby SA., et al. Correlation functions quantify super-resolution images and estimate apparent clustering due to over-counting. *PLoS One* 2012;7:e31457. 10.1371/journal.pone.0031457 PMC3288038 22384026

[75] Daims H, Lücker S, Wagner M. Daime, a novel image analysis program for microbial ecology and biofilm research. *Environ Microbiol* 2006;8:200–13. 10.1111/j.1462-2920.2005.00880.x 16423009

[76] Zheng J, Ge Q, Yan Y., et al. Dbcan3: automated carbohydrate-active enzyme and substrate annotation. *Nucleic Acids Res* 2023;51:W115–21. 10.1093/nar/gkad328 PMC10320055 37125649

[77] Zimmermann J, Kaleta C, Waschina S. Gapseq: informed prediction of bacterial metabolic pathways and reconstruction of accurate metabolic models. *Genome Biol* 2021;22:81. 10.1186/s13059-021-02295-1 PMC7949252 33691770

[78] Lieven C, Beber ME, Olivier BG., et al. Memote for standardized genome-scale metabolic model testing. *Nat Biotechnol* 2020;38:272–6. 10.1038/s41587-020-0446-y PMC7082222 32123384

[79] Zelezniak A, Andrejev S, Ponomarova O., et al. Metabolic dependencies drive species co-occurrence in diverse microbial communities. *PNAS.* 2015;112:6449–54. 10.1073/pnas.1421834112 PMC4443341 25941371

[80] Liu W, Jacquiod S, Brejnrod A., et al. Deciphering links between bacterial interactions and spatial organization in multispecies biofilms. *ISME J.* 2019;13:3054–66. 10.1038/s41396-019-0494-9 PMC6864094 31455806

[81] Bani A, Pioli S, Ventura M., et al. The role of microbial community in the decomposition of leaf litter and deadwood. *Appl Soil Ecol* 2018;126:75–84. 10.1016/j.apsoil.2018.02.017

[82] Wilcox A, Hall M, Menzies J., et al. Carboxymethyl cellulose biodegradation by activated sludge microbial communities. *Sci Total Environ* 2025;991:179942. 10.1016/j.scitotenv.2025.179942 40544631

[83] Yang N, Nesme J, Røder HL., et al. Emergent bacterial community properties induce enhanced drought tolerance in arabidopsis. *NPJ Biofilms Microbiomes* 2021;7:82. 10.1038/s41522-021-00253-0 PMC8602335 34795326

[84] Wilhelm RC, Singh R, Eltis LD., et al. Bacterial contributions to delignification and lignocellulose degradation in forest soils with metagenomic and quantitative stable isotope probing. *ISME J* 2019;13:413–29. 10.1038/s41396-018-0279-6 PMC6331573 30258172

[85] Shin S, Hyun J. Rheological properties of cellulose nanofiber hydrogel for high-fidelity 3d printing. *Carbohyd Polym* 2021;263:117976. 10.1016/j.carbpol.2021.117976 33858573

[86] Heise K, Kontturi E, Allahverdiyeva Y., et al. Nanocellulose: recent fundamental advances and emerging biological and biomimicking applications. *Adv Mater* 2021;33:e2004349. 10.1002/adma.202004349 PMC11468234 33289188

[87] Ee LY, Li SFY. Recent advances in 3d printing of nanocellulose: structure, preparation, and application prospects. *Nanoscale Adv* 2021;3:1167–208. 10.1039/d0na00408a PMC9418582 36132876

[88] Liu W, Russel J, Røder HL., et al. Low-abundant species facilitates specific spatial organization that promotes multispecies biofilm formation. *Environ Microbiol* 2017;19:2893–905. 10.1111/1462-2920.13816 28618083

[89] Wang CM, Shyu CL, Ho SP., et al. Characterization of a novel thermophilic, cellulose-degrading bacterium paenibacillus sp. strain b39. *Lett Appl Microbiol* 2008;47:46–53. 10.1111/j.1472-765X.2008.02385.x 18554262

[90] Ronin D, Hansen MF, Flaig ML., et al. Unfolding the collective functional potential of a synergistic multispecies community through genotypic and phenotypic analyses. *Biofilm.* 2025;10:100290. 10.1016/j.bioflm.2025.100290 PMC12246864 40657038

[91] Herschend J, Damholt ZBV, Marquard AM., et al. A meta-proteomics approach to study the interspecies interactions affecting microbial biofilm development in a model community. *Sci Rep-Uk* 2017;7:16483. 10.1038/s41598-017-16633-6 PMC5705676 29184101

[92] Nasipuri P, Herschend J, Brejnrod AD., et al. Community-intrinsic properties enhance keratin degradation from bacterial consortia. *PLoS One* 2020;15:e0228108. 10.1371/journal.pone.0228108 PMC6994199 32004342

[93] Hansen LBS, Ren D, Burmølle M., et al. Distinct gene expression profile of xanthomonas retroflexus engaged in synergistic multispecies biofilm formation. *ISME J.* 2017;11:300–3. 10.1038/ismej.2016.107 PMC5315472 27505346

[94] Watrous JD, Phelan VV, Hsu CC., et al. Microbial metabolic exchange in 3d. *ISME J.* 2013;7:770–80. 10.1038/ismej.2012.155 PMC3603389 23283018

[95] de Cárcer DA . Experimental and computational approaches to unravel microbial community assembly. *Comput Struct Biotec* 2020;18:4071–81. 10.1016/j.csbj.2020.11.031 PMC7736701 33363703

[96] van Hoogstraten SWG, Kuik C, Arts JJC., et al. Molecular imaging of bacterial biofilms-a systematic review. *Crit Rev Microbiol* 2024;50:971–92. 10.1080/1040841x.2023.2223704 PMC11523921 37452571

[97] Sauer K, Stoodley P, Goeres DM., et al. The biofilm life cycle: expanding the conceptual model of biofilm formation. *Nat Rev Microbiol.* 2022;20:608–20. 10.1038/s41579-022-00767-0 PMC9841534 35922483

[98] Obando MC, Serra DO. Dissecting cell heterogeneities in bacterial biofilms and their implications for antibiotic tolerance. *Curr Opin Microbiol* 2024;78:102450. 10.1016/j.mib.2024.102450 38422558

[99] Romaní AM, Fund K, Artigas J., et al. Relevance of polymeric matrix enzymes during biofilm formation. *Microb Ecol* 2008;56:427–36. 10.1007/s00248-007-9361-8 18227962

[100] Brethauer S, Shahab RL, Studer MH. Impacts of biofilms on the conversion of cellulose. *Appl Microbiol Biot* 2020;104:5201–12. 10.1007/s00253-020-10595-y PMC7275028 32337627

[101] Pontrelli S, Bigovic Villi K, Sichert A., et al. Degradation of extracellular polymeric substances shapes microbial community diversity. *PLoS Biol* 2025;23:e3003287. 10.1371/journal.pbio.3003287 PMC12306770 40729313

[102] Mitri S, Clarke E, Foster KR. Resource limitation drives spatial organization in microbial groups. *ISME J.* 2016;10:1471–82. 10.1038/ismej.2015.208 PMC5029182 26613343

[103] Khandoori R, Mondal K, Ghosh P. Resource limitation and population fluctuation drive spatiotemporal order in microbial communities. *Soft Matter* 2024;20:3823–35. 10.1039/d4sm00066h 38647378

[104] Pignon E, Schaerli Y. Deciphering microbial spatial organization: insights from synthetic and engineered communities. *Isme Commun* 2025;5:ycag107. 10.1093/ismeco/ycaf107 PMC12354948 40821451

[105] Doan HK, Leveau JHJ. Artificial surfaces in phyllosphere microbiology. *Phytopathology.* 2015;105:1036–42. 10.1094/Phyto-02-15-0050-Rvw 25894316

[106] Luís A, Domingues F, Ramos A. Production of hydrophobic zein-based films bioinspired by the lotus leaf surface: characterization and bioactive properties. *Microorganisms.* 2019;7:267. 10.3390/microorganisms7080267 PMC6723050 31426406

[107] Gomes LC, Saubade F, Amin M., et al. A comparison of vegetable leaves and replicated biomimetic surfaces on the binding of escherichia coli and listeria monocytogenes. *Food Bioprod Process* 2022;137:99–112. 10.1016/j.fbp.2022.11.003

[108] Douglas AE . The microbial exometabolome: ecological resource and architect of microbial communities. *Philos T R Soc B* 2020;375:20190250. 10.1098/rstb.2019.0250 PMC7133521 32200747

[109] Konopka A . What is microbial community ecology? *ISME J.* 2009;3:1223–30. 10.1038/ismej.2009.88 19657372

[110] Widder S, Allen RJ, Pfeiffer T., et al. Challenges in microbial ecology: building predictive understanding of community function and dynamics. *ISME J.* 2016;10:2557–68. 10.1038/ismej.2016.45 PMC5113837 27022995

[111] Martinez-Garcia R, Tarnita CE, Bonachela JA. Spatial patterns in ecological systems: from microbial colonies to landscapes. *Emerg Top Life Sci* 2022;6:245–58. 10.1042/Etls20210282 35678374

[112] Martinez-Rabert E, van Amstel C, Smith C., et al. Environmental and ecological controls of the spatial distribution of microbial populations in aggregates. *PLoS Comput Biol* 2022;18:e1010807. 10.1371/journal.pcbi.1010807 PMC9810174 36534694

[113] Oriano M, Zorzetto L, Guagliano G., et al. The open challenge of in vitro modeling complex and multi-microbial communities in three-dimensional niches. *Front Bioeng Biotech* 2020;8:539319. 10.3389/fbioe.2020.539319 PMC7606986 33195112

[114] Borer B, Or D. Spatiotemporal metabolic modeling of bacterial life in complex habitats. *Curr Opin Biotech* 2021;67:65–71. 10.1016/j.copbio.2021.01.004 33493977

[115] Bosi E, Bacci G, Mengoni A., et al. Perspectives and challenges in microbial communities metabolic modeling. *Front Genet* 2017;8:88. 10.3389/fgene.2017.00088 PMC5478693 28680442

[116] Bauer E, Zimmermann J, Baldini F., et al. Bacarena: individual-based metabolic modeling of heterogeneous microbes in complex communities. *PLoS Comput Biol* 2017;13:e1005544. 10.1371/journal.pcbi.1005544 PMC5460873 28531184

[117] Dukovski I, Bajić D, Chacón JM., et al. A metabolic modeling platform for the computation of microbial ecosystems in time and space (comets). *Nat Protoc* 2021;16:5030–82. 10.1038/s41596-021-00593-3 PMC10824140 34635859

[118] Ivleva NP, Kubryk P, Niessner R. Raman microspectroscopy, surface-enhanced raman scattering microspectroscopy, and stable-isotope raman microspectroscopy for biofilm characterization. *Anal Bioanal Chem* 2017;409:4353–75. 10.1007/s00216-017-0303-0 28389920

[119] Roder HL, Herschend J, Russel J., et al. Enhanced bacterial mutualism through an evolved biofilm phenotype. *ISME J.* 2018;12:2608–18. 10.1038/s41396-018-0165-2 PMC6194118 29977009

