# Supplementary material for: Carbon substrate type shapes spatial self-organization in a multi-species biofilm community
Source: ISME J. 2026 Aug 27;20(1):wrag223. doi: 10.1093/ismejo/wrag223 (PMC13617570; doi:10.1093/ismejo/wrag223)
Supplement: Supplementary file 1 [file supplementary_material_wrag223.zip › ISMEJ-D-26-00502_Supplementary-Information_wrag223.pdf]

Supplementary Information

Carbon substrate type shapes spatial self-organization in a multi-species biofilm community

Deyong Zhu[1] *, Anna J. Svagan[2], Nan Yang[1], Mads Frederik Hansen[1], Michael Kühl[3], Mette Burmølle[1] *

[1] Section of Microbiology, Department of Biology, University of Copenhagen, Copenhagen 2100, Denmark

[2] Department of Fibre and Polymer Technology, Royal Institute of Technology (KTH), SE-100 44 Stockholm, Sweden

[3] Marine Biology Section, Department of Biology, University of Copenhagen, Strandpromenaden 5, Helsingør 3000, Denmark

* Correspondence: deyong.zhu@bio.ku.dk (D.Z.); burmolle@bio.ku.dk (M.B.)

Supplementary materials for this article contain the following tables and figures:

Supplementary Methods

Fig. S1. Biofilm formation of the sum of monocultures versus the SynCom.

Fig. S2. Growth curve measurement on different carbon sources.

Fig. S3. Biofilm formation on different concentrations of carbon sources.

Fig. S4. Digital images of Congo Red binding assay.

Fig. S5. Cellulose degradation test with cellulose filter paper.

Fig. S6. Rheological property measurement of 3D-printing hydrogels.

Fig. S7. AFM image of the cellulose nanofibers (TEMPO-CNFs).

Fig. S8. SEM images of the surface of 3D printed artificial leaves.

Fig. S9. Averaged pair correlation analysis for spatial organization of the SynCom.

Fig. S10. Biofilm formation on conditioned culture supernatants.

Supplementary Methods

Growth curve measurement

Growth curves of the mono- and quadruple-cultures in M9 supplemented with different carbon sources (**Table S2**) were measured using a plate reader (Synergy H1, BioTek Instrument, VT, USA) at 24 °C. 96-well microtiter plates containing bacterial cultures were prepared as described previously in the CV assay. Each sample contained three technical replicates.

Cellulose degradation test on filter paper

The examination of pure cellulose degradation was conducted using porous filter paper (Whatman paper, Z695068). An aliquot of 300 µL of monocultures and the SynCom culture (with OD_{600} adjusted to 0.3) was inoculated on the autoclaved filter paper strips in each tube, individually. After 6 hrs to allow the bacterial cells to sediment onto the paper's surface, 5 mL minimum media M9 was added to each tube, and all tubes were incubated at 24 °C with continuous monitoring for up to 14 days. Blank filter paper strips with no inoculation were used as control. Each sample contained three technical replicates.

Biofilm formation experiments on culture supernatants

The supernatants of *P. amylolyticus* (P) and the SynCom were prepared from cultures on different carbon sources in M9 media with cellobiose, cellobiose+xylan, and cellobiose+CMC, respectively. The inoculate OD was adjusted to 0.15 for *P. amylolyticus* and at equal mixing ratio for the SynCom of PMSX. The supernatants were harvested after 16-hour incubation at 24 °C with shaking at 250 rpm, followed by centrifugation at 10,000 rpm for 20 min twice and filtration with 0.22 µm filters. The total biomass of mono-biofilm growth on the prepared culture supernatants was measured by the CV assay as described in the section of Materials and Methods. Finally, the absorbance of CV in ethanol was measured at 590 nm by a plate reader (ELx808, BioTeck Instrument, VT, USA). Each sample contained three technical replicates.

Atomic Force Microscopy (AFM) characterization

The AFM micrograph of TEMPO-CNF was obtained using a MultiMode 8 (Bruker, Santa Barbara, CA, USA) in PeakForce QNM mode using ScanAsyst cantilevers (Bruker, Camarillo, CA, USA). The silicon wafers were first treated by adsorbing a layer of Polyethylenimine (PEI, 0.01 wt%, 1 min), followed by rinsing with Milli-Q water and drying. A drop of the TEMPO-CNF suspension (0.01 wt%) was deposited on the wafer, followed by immediate rinsing by MilliQ-water and drying.

Scanning Electron Microscopy (SEM) characterization

The morphology of 3D-printed substrates comprising different carbon sources was characterized by SEM. Briefly, the hydrogel-based substrates were pretreated by immersion into liquid nitrogen until completely frozen, followed by lyophilization to preserve the structure. The lyophilized samples were sputter-coated with gold and multiple micrographs were obtained at selected regions using a Quanta 200 environmental scanning electron microscope (FEI, OR, USA). Images at 300x magnification, 5 kV were presented to demonstrate the microscale porous structure.

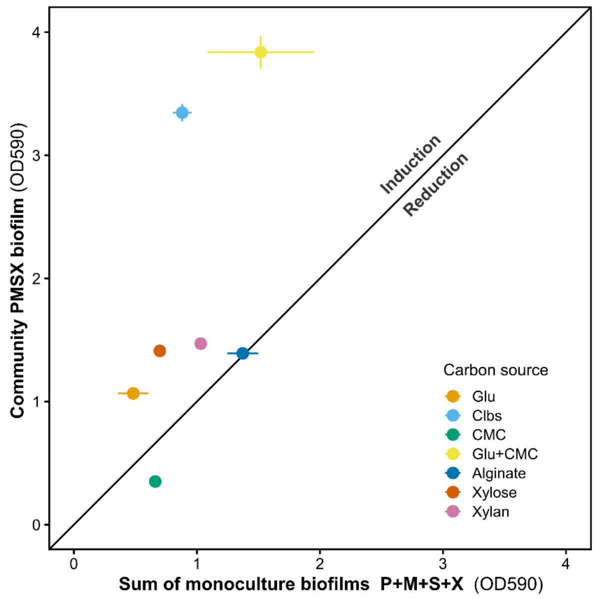

Fig. S1. Monoculture versus SynCom (Community PMSX) biofilm formation under static conditions using crystal violet to assess biomass production after 24 hours of growth in different carbon sources. Data points above the line represent the SynCom that are performing better than the sum of monoculture biofilms. P: *P. amylolyticus*, M: *M. oxydans*, S: *S. rhizophila*, and X: *S. maltophilia*.

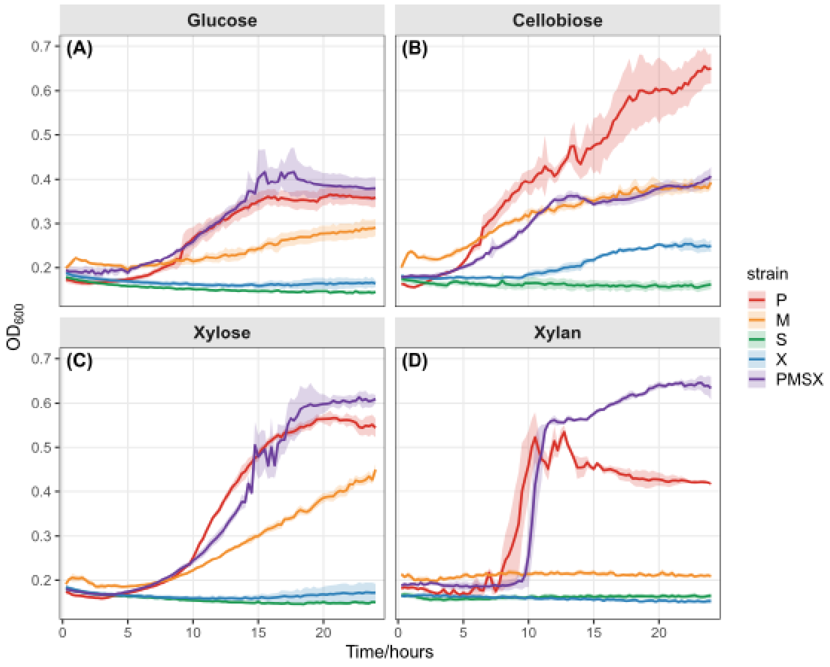

Fig. S2. Growth curves in the M9 medium with different carbon sources. (A) Glucose, (B) Cellobiose, (C) Xylose, and (D) Xylan. Each plot represents three technical replicates. P: *P. amylolyticus*, M: *M. oxydans*, S: *S. rhizophila*, and X: *S. maltophilia*.

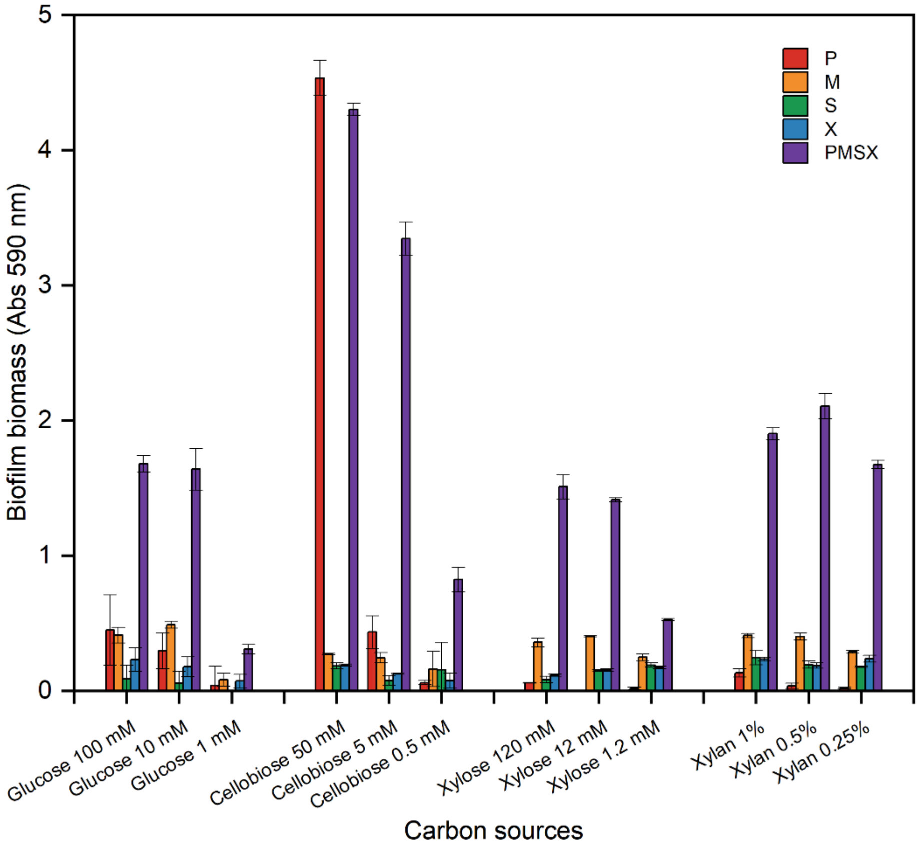

Fig. S3. Screening of different-concentration carbon sources by the crystal violet (CV) assay for mono- and multi-species cocultured biofilm formation. P: *P. amylolyticus*, M: *M. oxydans*, S: *S. rhizophila*, and X: *S. maltophilia*.

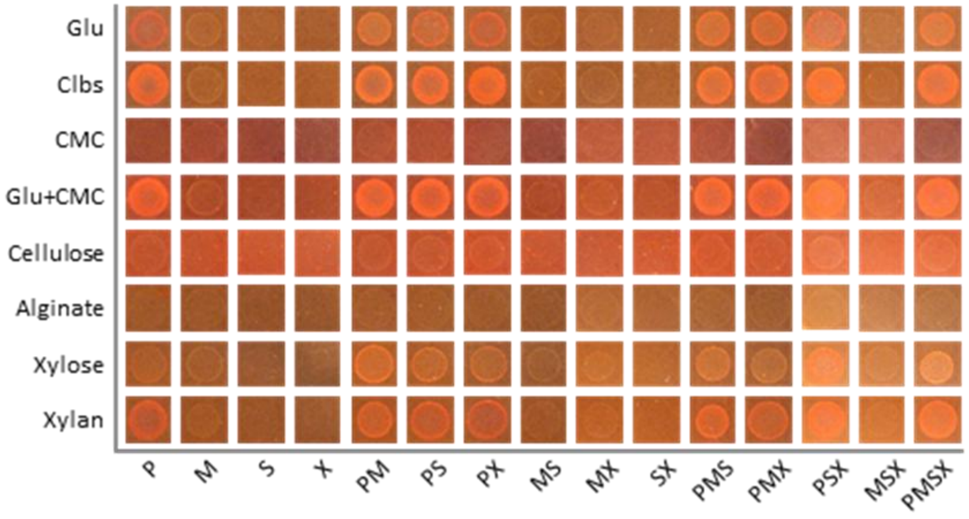

Fig. S4. Congo red staining plates with different carbon sources showing mono-, dual-, triple-, and quad-species biofilm colonies. Glu: glucose; Clbs: cellobiose; CMC: carboxymethyl cellulose; Glu+CMC: glucose with carboxymethyl cellulose. P: *P. amylolyticus*, M: *M. oxydans*, S: *S. rhizophila*, and X: *S. maltophilia*.

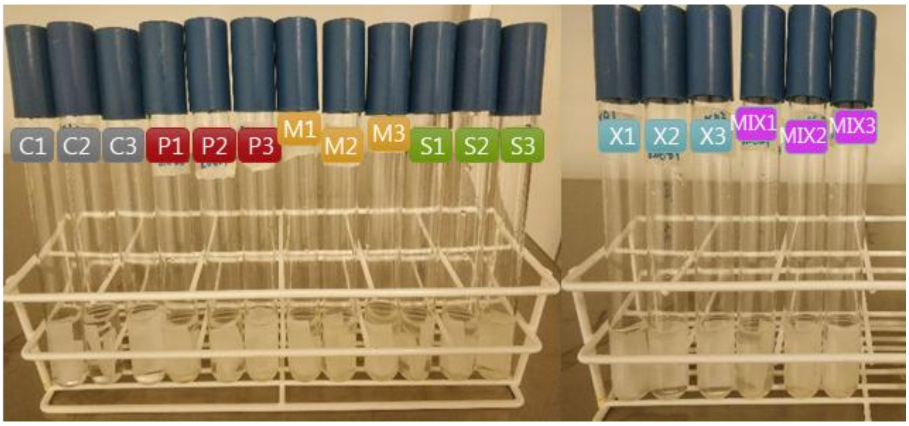

Fig. S5. Images of cellulose degradation test with porous filter paper. Continuous monitoring up to 14 days revealed no observable change either in the media or on the filter paper. C: Control without inoculating any bacteria, P: *P. amylolyticus*, M: *M. oxydans*, S: *S. rhizophila*, X: *S. maltophilia*, and MIX: the full consortium. Three replicates of each.

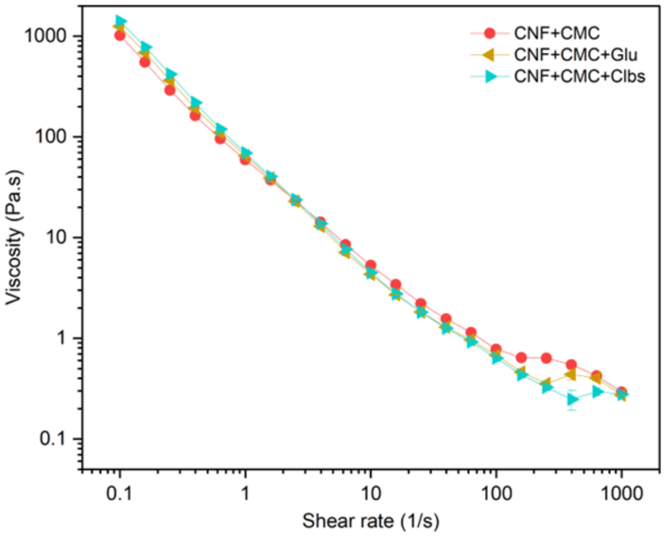

Fig. S6. Rheological property measurement of 3D printing materials. CNF: *2,2,6,6*-tetramethylpiperidine-*1*-oxyl-cellulose nanofiber; CMC: carboxymethyl cellulose; Glu: glucose; Clbs: cellobiose. Each data point represents three technical replicates.

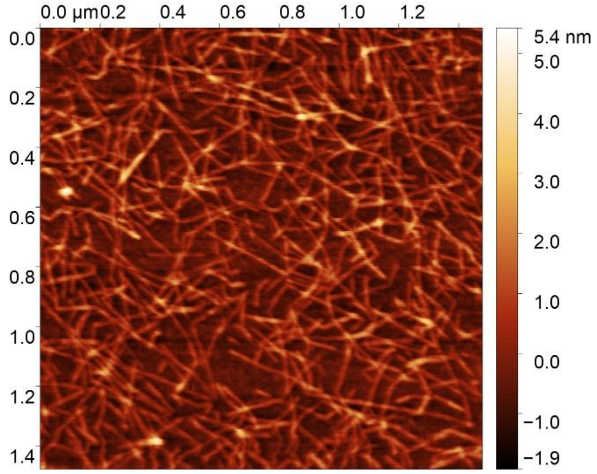

Fig. S7. Atomic force microscopy (AFM) imaging showing the size of the prepared CNF (TEMPO-cellulose nanofiber).

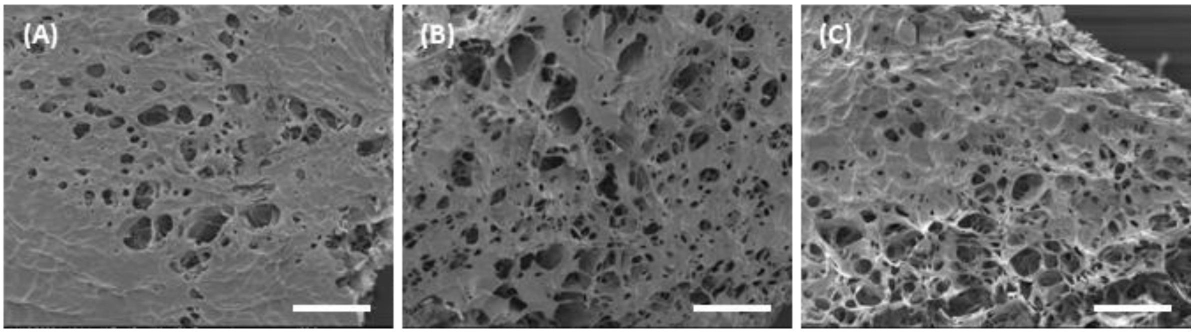

Fig. S8. Images by scanning electron microscopy (SEM) demonstrating the interconnected porous properties of printed artificial leaves with carbon sources of (A) CNF, (B) CNF+CMC, and (C) CNF+Xylan. Scale bar: 200 µm. The hydrogels were freeze-dried prior to imaging.

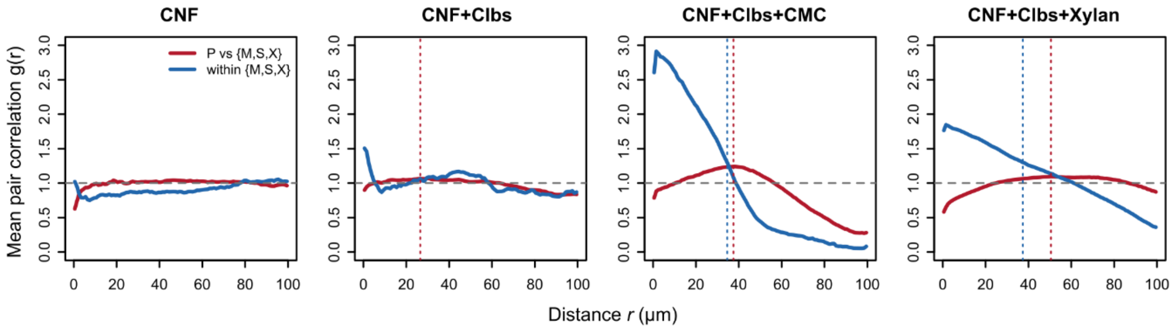

Fig. S9. Pair-correlation functions *g(r)* computed from segmented FISH images of the four-member community grown on four substrates. For each condition, single-channel images

were Otsu-thresholded into binary masks and *g(r)* was obtained by FFT-based radial cross-correlation, normalized to the co-imaged field so that g(r) = 1 denotes a random spatial arrangement (grey dashed line), g(r) > 1 co-localization (cells found together more than expected), and g(r) < 1 segregation (cells apart). In each panel the red curve is the mean *g(r)* of the putative primary degrader P against the other three members, and the blue curve is the mean *g(r)* among the non-degrader members, each averaged over its three constituent strain pairs. Vertical dotted lines mark the domain size and the core radius (Red; the distance of maximal P-vs-rest correlation; Blue: the distance of maximal M/S/X correlation, where present;). On the simple substrates (e.g. cellobiose) both curves stay near 1, indicating a well-mixed, unstructured community; while on the polymeric substrates (e.g. CMC, xylan) the non-degrader members show strong short-range co-clustering that decays over tens of micrometers, while *P. amylolyticus* is enriched at the periphery of those clusters which is consistent with substrate complexity driving spatial self-organization around *P. amylolyticus*.

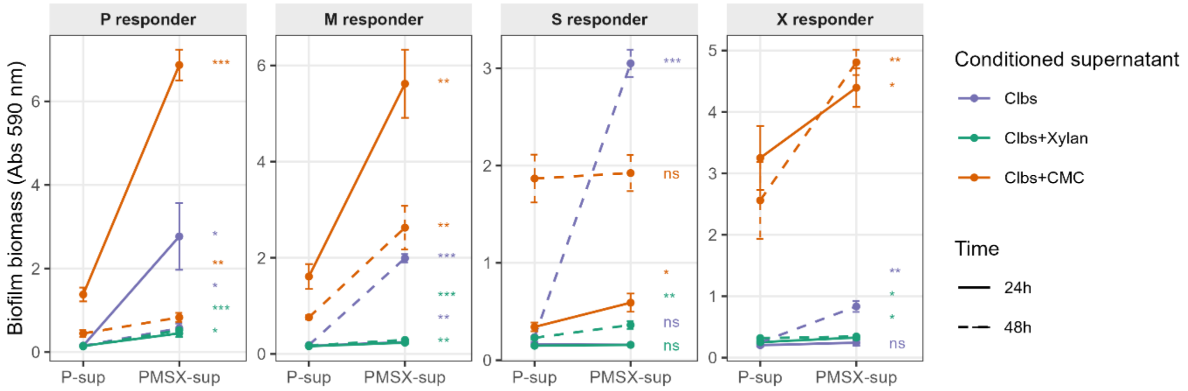

Fig. S10. Biofilm formation comparison of individual SynCom members on community (PMSX)-conditioned *versus* P-conditioned supernatants. Each strain (P, M, S, X; responder) was grown on conditioned supernatants prepared from either *P. amylolyticus* monoculture (P-sup) or the SynCom community (PMSX-sup) on three carbon sources (cellobiose (Clbs), Clbs+xylan, Clbs+CMC), and biofilm biomass was quantified by the CV assay at 24 h (solid line) and 48 h (dashed line). Points and error bars are mean ± SD of three technical replicates. Asterisks indicate the increase from P-sup to PMSX-sup within each condition, processed by R using one-sided Welch's *t*-test: *$p < 0.05$, **$p < 0.01$, ***$p < 0.001$; ns, not significant. For each SynCom member, biofilm formation in PMSX-conditioned supernatants exceeded that in P-conditioned supernatants in the majority of conditions. P: *P. amylolyticus*, M: *M. oxydans*, S: *S. rhizophila*, and X: *S. maltophilia*.
